# A comprehensive well-to-wake climate impact assessment of sustainable aviation fuel

**DOI:** 10.1038/s41598-025-13445-x

**Published:** 2025-08-30

**Authors:** Luc Boerboom, Arvind Gangoli Rao, Volker Grewe, Feijia Yin

**Affiliations:** 1 Faculty of Aerospace Engineering, Delft University of Technology, Delft, The Netherlands; 2 Deutsches Zentrum für Luft- und Raumfahrt, Institut für Physik der Atmosphäre, Oberpfaffenhofen, Germany

**Keywords:** Climate change, Engineering

## Abstract

The aviation industry and policymakers are advocating Sustainable Aviation Fuels (SAF) as one of the main pillars for making the aviation industry sustainable. However, regulatory frameworks like CORSIA and the EU Renewable Energy Directive often exclude the climate impact from in-flight non-CO_2_ emissions (e.g., NO_x_, H_2_O, and soot emissions), which is important in determining the effect of SAF in reducing the climate impact of aviation. To bridge this gap, we evaluate the total global warming effects of SAF from a well-to-wake analysis, which includes the climate effects from CO_2_ emissions of the well-to-wake combined with the non-CO_2_ emissions of the pump-to-wake (i.e., inflight). We quantify the climate impact of NO_x_, H_2_O and contrails and convert them to a CO_2_ equivalence (CO_2_e) factor based on a climate metric, for instance, the Average Temperature Response over a given time horizon (i.e., 20, 50 and 100 years). The resulting well-to-wake CO_2_e values for SAF vary from about 150 to 250 g/MJ, depending on the specific fuel pathways. Our analysis shows that the maximum reduction in CO_2_e emissions when using SAF is less than 50% compared to conventional jet fuel, mainly due to the inflight NO_x_ and contrail effects.

**Supplementary Information:**

The online version contains supplementary material available at 10.1038/s41598-025-13445-x.

## Introduction

Aviation is an important economic sector that provides a fast and reliable means of transportation. However, in 2018, the sector was responsible for the emission of almost 1 Gt CO_2_, equivalent to 2.4% of global anthropogenic CO_2_ emissions including land use change^1^. Lee et al. (2021) estimated that the share of anthropogenic effective radiative forcing from aviation was around 4%, including both CO_2_ and non-CO_2_ effects^1^.

The non-CO_2_ climate impact stems primarily from the releases of nitrogen oxides (NO_x_), water vapour (H_2_O), and particle emissions. These non-CO_2_ effects have strong spatial and temporal dependencies and are associated with a high degree of uncertainty^1,2^. NO_x_ emissions can lead to positive radiative forcing (warming effect) as they serve as a precursor for short-term ozone (O_3_) production but also cause a cooling effect as they destroy background methane (CH_4_) and the associated ozone (named as Primary Mode Ozone, PMO)^3,4^. Water vapour has a negligible greenhouse gas (GHG) effect when emitted in the lower levels of the troposphere due to its short lifetime, but when emitted at high altitudes (near the tropopause or in the stratosphere), its GHG effect becomes stronger due to its increased residence time^5^. Soot and sulfur particulate emissions have relatively small direct climate effects, but when H_2_O condenses onto these particles and freezes to form a contrail, this can lead to a significant positive forcing, especially at night^6^. In total, the non-CO_2_ effects represented roughly two-thirds of total aviation’s radiative forcing in 2018 considering the best estimate^1^.

One way to reduce aviation’s climate impact is by using sustainable aviation fuel (SAF)^7^. For instance, aircraft operators are allowed to use SAF to reduce their carbon offsetting requirements through the Carbon Offsetting & Reduction Scheme for International Aviation (CORSIA) and the EU Emissions Trading Scheme (ETS)^8^. The carbon footprint of SAFs is often quantified in grams of CO_2_ equivalence per megajoule (gCO_2_ e/MJ) by means of a Lifecycle Analysis (LCA), which allows the user to identify the environmental benefit of SAF compared to Conventional Jet Fuel (CJF)^9–11^. Based on these findings, a certain type of SAF can be promoted or discouraged by decision-makers. As mandated by regulatory frameworks, e.g., CORSIA, the Renewable Energy Directive (RED) and the Low Carbon Fuel Standard (California), the current LCAs focus mainly on CO_2_, CH_4_ and N_2_O emissions produced from Well to Wake (WtW). The WtW scope is composed of two stages: (1) fuel production and distribution (Well-to-Pump (WtP)) and (2) fuel combustion (Pump-to-Wake (PtW)). Accordingly, 100% SAF has the potential to reduce the lifecycle GHG emissions by up to 94% compared to CJF, depending on the feedstocks and technology pathways, e.g., carbon capture^11,12^.

When it comes to the PtW non-CO_2_ emissions, burning SAF can reduce soot particles and thereby reducing contrail radiative forcing^13^. Meanwhile, SAF has little effect on the NO_x_ emissions^14^. Therefore, it is necessary to include the climate impact of the non-CO_2_ emissions from burning SAF when quantifying the climate impact benefits of SAF. The previous work^15,16^ analyzed the inflight non-CO_2_ climate effects of SAF using the Global Warming Potential (GWP) with horizons of 20-100-500 years. It was concluded that the incorporation of these non-CO_2_ effects reduced the relative merit of using SAF, as SAF could not mitigate the non-CO_2_ climate impact. However, the analysis did not consider the fact that SAF is able to reduce the contrail climate impact^17,18^ because of the reduced soot emissions compared to CJF^19,20^. Moreover, recent work from Megill et al.^21^ tested climate metrics against requirements, such as the consistency between the climate impact evaluation based on a requirement and a scenario analysis. The analysis showed that Average Temperature Response (ATR) as a climate metric was better suited for aviation than, e.g. GWP.

In this study, we perform the Life Cyle Climate Impact Analysis (LCCIA) to evaluate the climate benefit of SAF. To do so, we quantify the climate impact of NO_x_, H_2_O and contrails from the PtW stage and convert them to the gCO_2_e/MJ value based on two climate metrics, GWP and ATR, respectively for time horizons of 20-50-100 years^22,23^. The resulting gCO_2_e/MJ values are then combined with the Lifecycle GHG emissions of the CORSIA Eligible Fuels to obtain the overall WtW GHG emissions of SAF^24^. Accordingly, we reflect on environmental benefits of SAF and show in the discussion that the GHG emission reductions can be increased by allocating SAF to long range flights. The overall approach includes an integrated modelling chain of emission inventory generation, climate impact calculation and Monte Carlo simulations. The details are described in the method section.

## Results

### Climate impact of inflight emissions for CJF and SAF

**Fig. 1:**
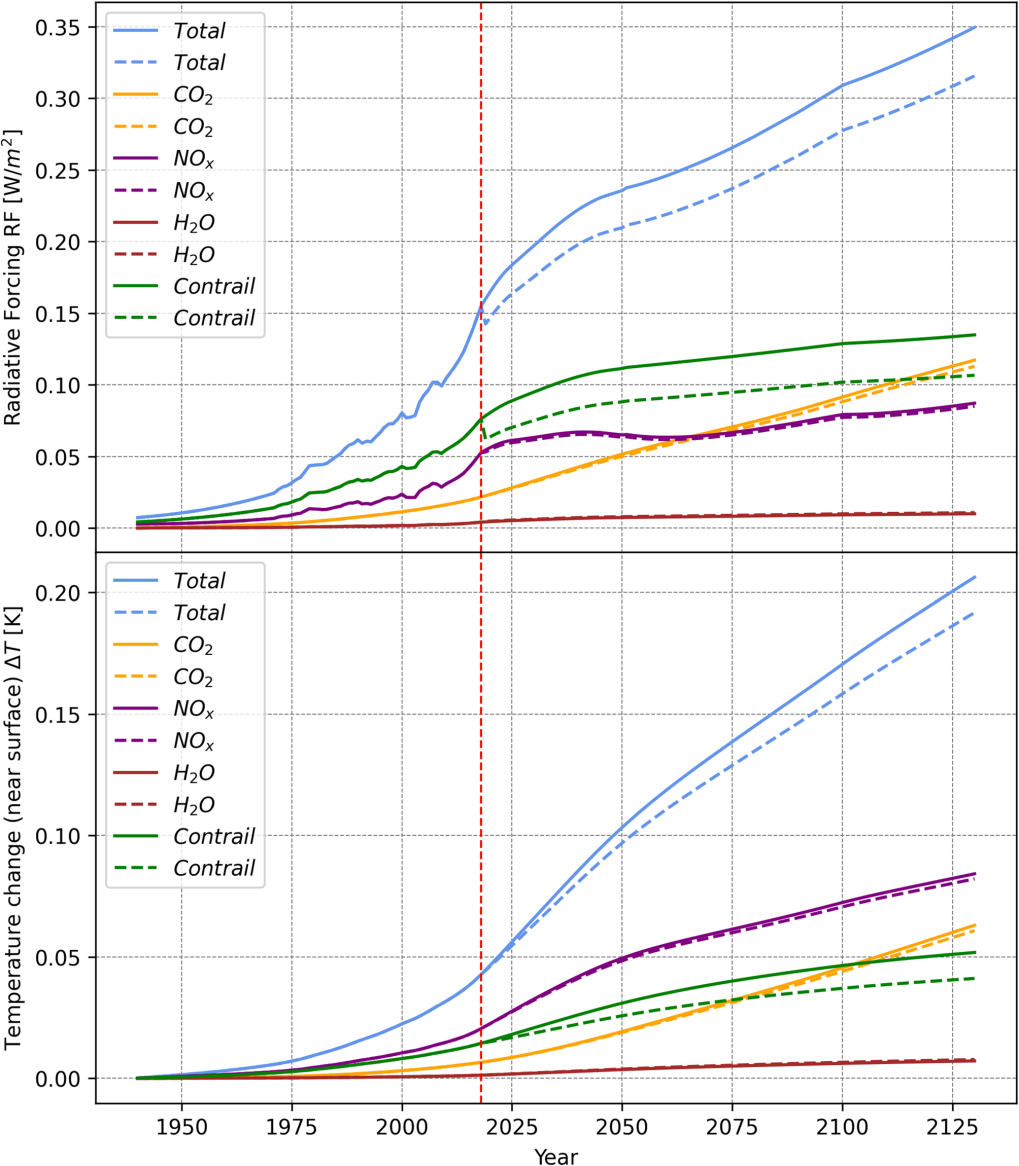
Radiative Forcing (RF) and temperature change responses (∆T) resulting from global aviation in-flight CO_2_ and non-CO_2_ emissions for 100% Conventional Jet Fuel (CJF) (solid lines) and 100% Sustainable Aviation Fuels (SAF) (dotted lines) from 1940–2130 for the business as usual (BAU) scenario. For SAF, the analysis does not include the reduction in Lifecyle CO_2_. Business as usual is a fleet forecast scenario where the fleet growth is predicted based on the market demand and meanwhile the efficiency improvements in the aircraft engine system and the operational aspects are considered.

Figure 1 shows the radiative forcing (RF) and the resulting near surface temperature change (∆T) caused by in-flight CO_2_, NO_x_, H_2_O emissions and contrails from the beginning of aviation to roughly 100 years after introduction of considerable amount of SAF (i.e., 1940–2130) for two cases: (1) 100% CJF (solid line) and (2) 100% SAF (dotted line). Case (1) assumes all aircraft use CJF from 1940 to 2130. For case (2), all aircraft use 100% CJF from 1940 to 2018, and in 2019 a perturbation is introduced that all aircraft use 100% SAF until 2130. The emission indices and calorific values of CJF and SAF are given in Table 1. The emission index of CO_2_ for SAF is based on the emissions at the engine tailpipe and does not include the reduction in Lifecyle CO_2_ in order to evaluate the CO_2_ equivalence for non-CO_2_ effects. Furthermore, the change in contrail RF for SAF in Table 1 is due to the reduction of soot number particles and is derived using the methodology from Grewe et al. (2021)^25^. The GHG emissions in Table 1 considers the amount of CO_2_ from combustion process. The RF and ∆T in Fig. 1 do not consider WtP emissions from fuel production and distribution. From 2019 onwards, the implementation of SAF will result in lower fuel consumption because of the higher calorific values of the fuel, which slightly reduces CO_2_, NO_x_, and soot emissions for given emission indices.

**Table 1:** Settings used for the 100% CJF and 100% SAF scenarios concerning only the fuel combustion process. The EIco_2_ for SAF does not include the lifecyle CO_2_. $\:\varDelta\:RF$ refers to the changes of contrail radiative forcing with respect to the conventional jet fuel due to the reduction of soot particle numbers. The scaling of RF is based on literature^25^.

Fuel type	CJF	SAF
CO_2_ emission index, EI_CO2_ [kg_CO2_/kg_fuel_]	3.16	3.10
Water emission index, EI_H2O_ [kg_H2O_/ kg_fuel_]	1.25	1.38
Fuel calorific value [MJ/kg]	43.1	44.2
Greenhouse Gas emissions [g_CO2_/MJ]	73.3	70.2
∆RF of contrail [%]	0%	-20.9%

Furthermore, the CO_2_ emission index (EI_CO2_) of SAF is lower than CJF, hence reducing the total CO_2_ emission, the corresponding RF and ∆T. Due to the increased H_2_O emission index (EI_H2O_) for SAF, the RF and ∆T related to H_2_O emissions for SAF are slightly higher. The NO_x_ emission index (EI_NOx_) is the same for both fuels, as NO_x_ emissions are mainly affected by the engine architecture and combustion technology not the fuel itself^14^. The contrail RF of SAF is reduced by about 21% in 2130, mainly due to the reduced soot numbers of burning SAF. The reduction of soot number reduces the contrail lifetime, optical properties, and hence the climate impact^18^. More details about the RF derivations for SAF are given in Supplementary Table 2 and Supplementary Table 3. The Supplementary Table 4 compares the change of RF using SAF from different literatures and shows a large variation range from 20% − 50% depending on the model setups. While the current analysis falls into the variation range, further research is required to reduce the uncertainty of using SAF on the contrail radiative forcing.

### CO_2_ equivalent factors for CJF and SAF

Based on the temporal evolution of ∆T in Fig. 1, the ATR_*i*,H_ (average temperature response over a time horizon of H for an individual climate specie, *i*) is obtained. Accordingly, the CO_2_ equivalent (CO_2_e) factors of individual species as a ratio to the ${\text{ATR}_{\text{CO}_{2},\text{H}}}$ is derived (see Eq. (1)). These CO_2_e factors indicate the significance of non-CO_2_ effects on the increase of the average surface temperature compared to the CO_2_ emissions by CJF and SAF, respectively.

1 $$\:{CO}_{{2e,\:all,\:\:ATR}_{H}}=\:\frac{{\sum\:}_{i}{\int\:}_{2019}^{2019+H}{\varDelta\:T}_{i}\left(t\right)-{\varDelta\:T}_{i}\left(2019\right)\:dt}{{\int\:}_{2019}^{2019+H}{\varDelta\:T}_{{CO}_{2}}\left(t\right)-{\varDelta\:T}_{{CO}_{2}}\left(2019\right)\:dt}\:\:\:\:\:\:\:\:\:\:\:\:\:\:\:\:\:\:\:\:\:\:\:\:\:\:\:\:\:\:\:\:\:\:\:$$

In Eq. (1), H is the time horizon. The subscript *i* denotes the climate species of NO_x_, H_2_O, CO_2_ and contrail, respectively. The temperature change in 2019 caused by species *i* emitted by global aviation before 2019 (i.e.,$\:{\varDelta\:T}_{i}\left(2019\right)$) is subtracted to eliminate the temperature change that already has happened before 2019.

**Fig. 2:**
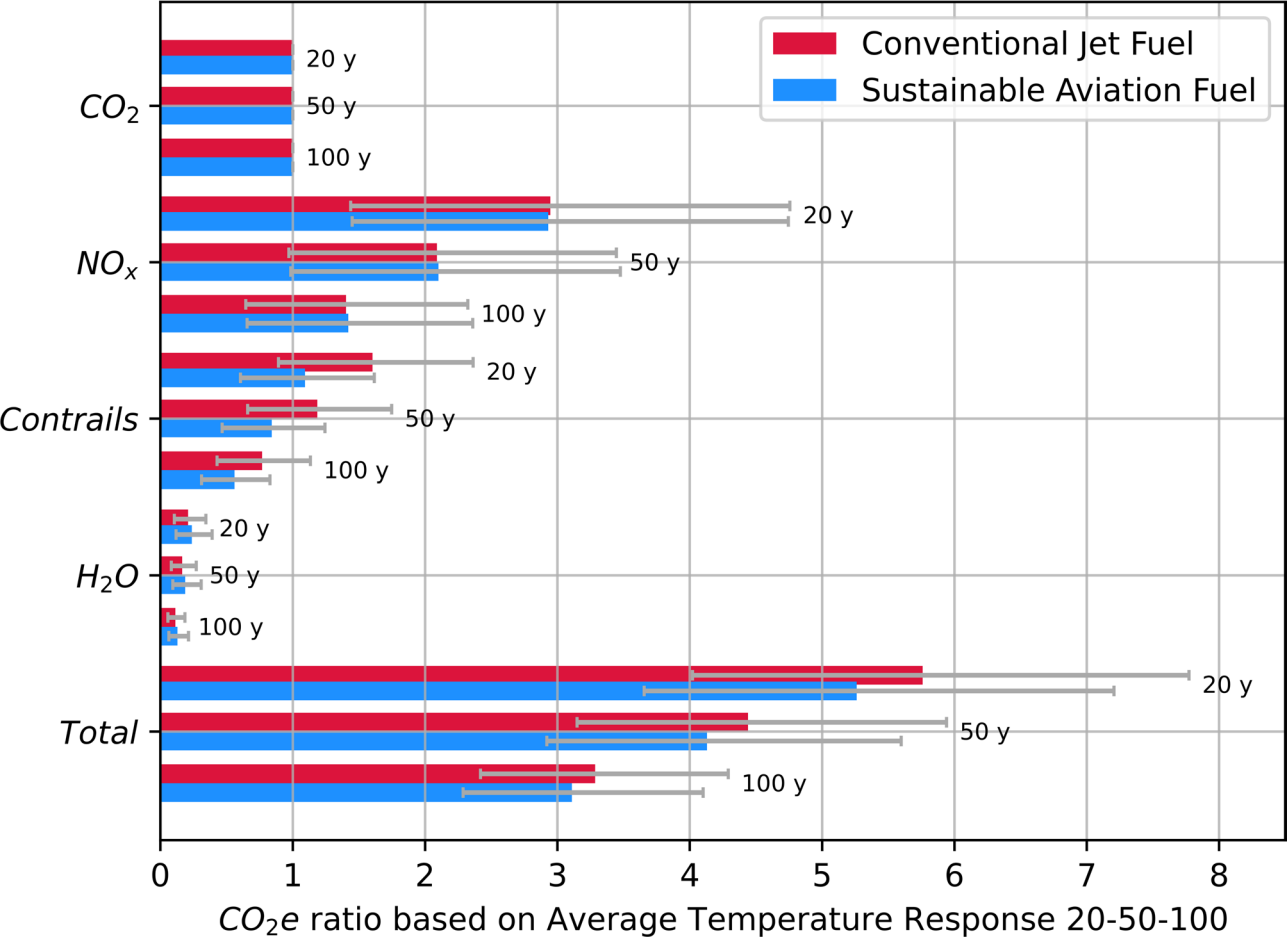
CO_2_e ratios of CO_2_, NO_x_, contrails and H_2_O based on the Average Temperature Response (ATR) over 20, 50 & 100 years calculated with Eq. (1) and derived from Fig. 1. Coloured bars represent the average, and uncertainty bars denote the 5th & 95th percentiles coming from a Monte Carlo analysis with 2000 simulations. The CO_2_e ratios are also given in tabulated form in Supplementary Table 6.

In Fig. 2, the CO_2_e ratios of all individual climate agents for SAF and CJF are presented for three different time horizons of 20, 50 and 100 years. For the shorter time horizon, the CO_2_e is larger since non-CO_2_ effects have a stronger short-term climate impact. As the time horizon increases, CO_2_ emissions build up and the resulting warming effect becomes more prominent over the long term. Hence, the total CO_2_e ratios reduce.

CO_2_e ratios based on the GWP have also been calculated following the similar approach of Eq. (1), but based on the time integrated RF. The consequent CO_2_e ratios indicate how much radiation (stratospherically adjusted RF) is forced back by non-CO_2_ effects compared to CO_2_. The results for CO_2_e ratios based on GWP are present in Supplementary Fig. 4 and Supplementary Table 7 and agree well with available literature^15^ (see Supplementary Table 8 and Supplementary Table 9 for more details).

### Well-to-wake CO_2_e emissions results

The well-to-wake CO_2_e emissions consist of two contributions: (1) the lifecycle CO_2_ emissions (production, distribution and combustion); and (2) the inflight non-CO_2_ emissions (PtW). In Table 1, the amount of CO_2_ emitted per MJ of fuel due to combustion are 73.3 gCO_2_/MJ for CJF and 70.2 gCO_2_/MJ for SAF. By multiplying these values with CO_2_e ratios in Fig. 2, the PtW CO_2_e emissions for the inflight non-CO_2_ effects can be derived. As a result, the PtW CO_2_e emission of CJF, due to the non-CO_2_ climate effects, is 167.9 gCO_2_e/MJ (based on the ATR100). Similarly, the PtW CO_2_e emissions for the non-CO_2_ effects of SAF is 147.7 gCO_2_e/MJ.

The PtW CO_2_e emissions of SAF for the non-CO_2_ effects can be flexibly included to any existing LCAs to obtain WtW CO_2_e emissions of SAF (see Eq. (2)). These novel WtW CO_2_e values thus represent the lifecycle climate impact in the format of gCO_2e_/MJ.

2 $$\:{\left[\frac{g{CO}_{2}e}{MJ}\right]\:}_{WtW}=\:{\left[\frac{g{CO}_{2}e}{MJ}\right]\:}_{WtP}+\:{\left[\frac{g{CO}_{2}e}{MJ}\right]\:}_{PtW}\:\:\:\:\:\:\:\:\:\:\:\:\:\:\:\:\:\:\:\:\:\:\:\:\:\:\:$$

**Fig. 3:**
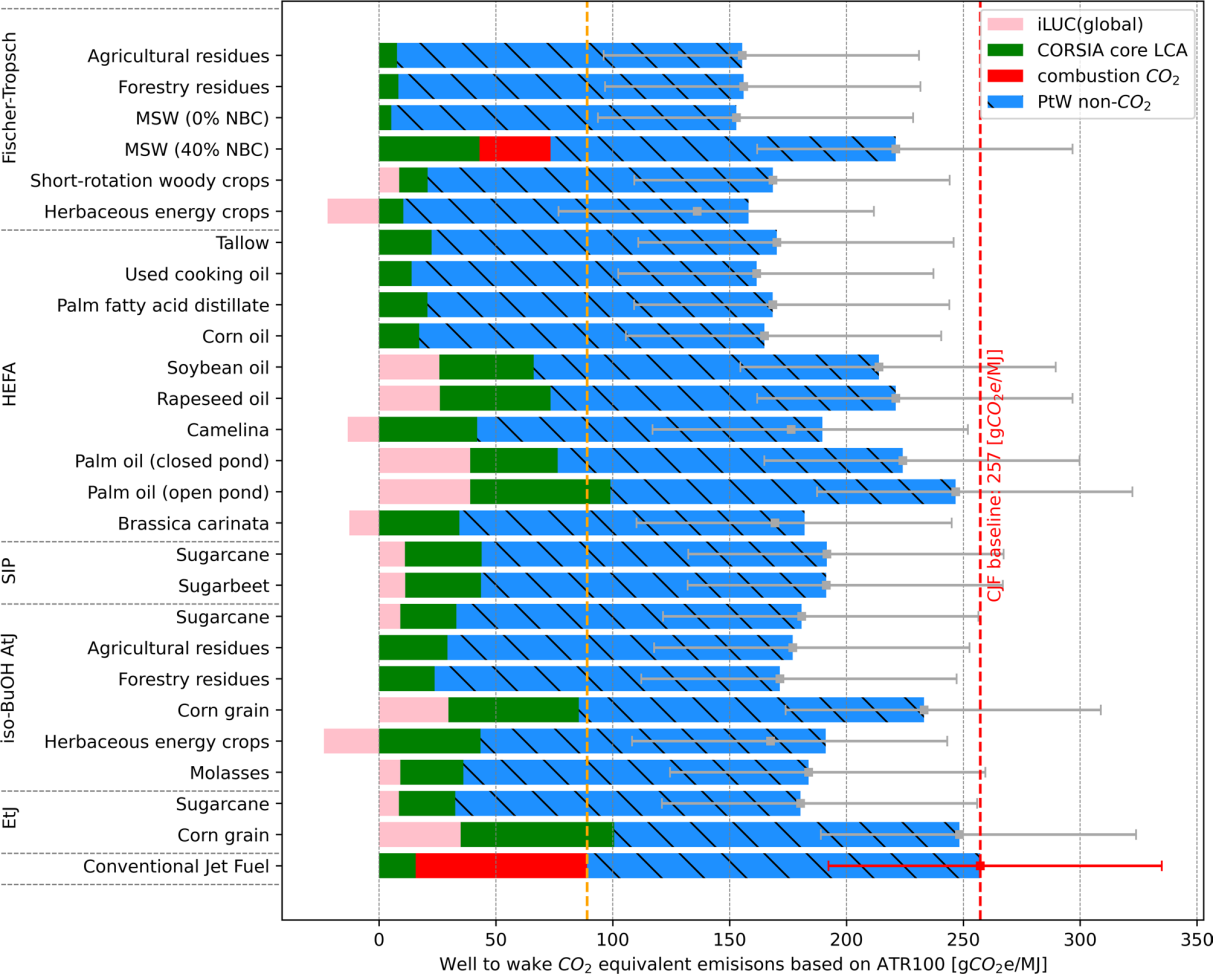
The estimated well-to-wake LCCIA CO_2e_ emissions in gCO_2_e/MJ based on the core lifecycle emissions values for CORSIA Eligible Fuels and conventional jet fuel^24^. Induced Land Use Change values (iLUC in pink) are the global values taken from CORSIA^24^. The PtW non-CO_2_ contribution is based on the ATR100. Error bars denote 5–95% confidence intervals based on a Monte Carlo analysis with 2000 simulations. NBC = Non-Biogenic Carbon. MSW = Municipal Solid Waste. HEFA = Hydrotreated Esters and Fatty Acids. SIP = Synthesized Iso-Paraffins. AtJ = Alcohol to Jet. EtJ = Ethanol to Jet.

Figure 3 shows a set of WtW CO_2e_ emissions considering the ICAO CORSIA eligible fuels and the CJF based on ATR100. The CORSIA core LCA emissions (in green) are taken from^24^. The Induced Land Use Change values (referring to the additional land used for fuel production, in pink).

Upon the inclusion of PtW non-CO_2_ effects, the equivalent CO_2_ emissions per MJ for CJF increases from 89 (the CORSIA LCA value, orange dotted line) to 256.9 [192.3-334.9] gCO_2_e/MJ (red dotted line). The PtW CO_2_e of SAF for the non-CO_2_ effects is about 147.7 g per MJ (blue bars in Fig. 3), which is slightly less than CJF (167.9 gCO_2_e/MJ). This indicates that the relative merit of using SAF increases mainly due to the reduction of contrail’s climate impact. For a shorter time horizon of 20- or 50- years, the CO_2_e ratios shown in Fig. 2 increase considerably, driven by the non-CO_2_ climate effects. Namely, using these shorter time horizons increases the non-CO_2_ emission reduction potential from SAF.

Furthermore, it is observed that some pathways like the palm oil (open pond) HEFA and corn grain EtJ, which previously had higher lifecycle emissions than CJF, now have lower WtW CO_2_e emissions than CJF. This is mainly because SAF contains less aromatics and decreases non-CO_2_ climate impact, i.e. the contrail impacts. It should be noted that in the RED (the legal framework used for the quantification of emissions reductions in the EU ETS), the standards set for a fuel to be classified as SAF are different. Nevertheless, the PtW values derived in this analysis are still applicable. We find that the intrapathway uncertainty increases which is aligned with the findings in^15^. If GWP based CO_2_e ratios were used instead of ATR based CO_2_e ratios to calculate the PtW results, the intrapathway uncertainty would decrease, as there is more uncertainty associated with calculating temperature change responses than radiative forcing responses.

While having one set of CO_2_e ratios for the entire fleet is convenient, it is not entirely fair for flights. The flights at higher cruise altitudes usually have a higher non-CO_2_ climate impact contribution than flights with lower cruise altitudes^26^. Therefore, flight-distance dependent CO_2_e factors are calculated for the clusters defined in Table 2 based on (great circle) flight distances. For each group, the ATR100 per climate species are calculated, and accordingly, cluster-specific CO_2_e ratios are derived for both CJF and SAF (the values are presented in the Supplementary Fig. 5). The main takeaway is that for the shorter-range flights, the climate impact reduction attained with SAF reduces compared to the long-range flights. The main reason is that short flights hardly reach contrail-forming altitudes. Therefore, the lower soot emissions from SAF will not benefit for contrail climate impact.

**Table 2:** Clusters of flights based on great circle distances in nautical miles.

Flight category	Flight distances (nautical miles)
Regional	< 300
Short	[300, 1000]
Medium	[1000, 2000]
Long	[2000, 4000]
Very long	> 4000

## Discussion

In this work, the PtW CO_2_e emissions based on the non-CO_2_ climate effects from NO_x_, H_2_O and contrails are newly derived and are combined with typical ICAO CORSIA LCA emissions to obtain the WtW CO_2_e emissions. Considering different climate metrics, the final LCCIA WtW varies. An example of considering the ATR100 metric shows that CJF has a WtW CO_2_e emissions of about 256.9 g per MJ. The LCCIA WtW for SAF varies from about 150 to 250 gCO_2_e/MJ depending on the exact SAF pathways. Furthermore, non-CO_2_ effects contribute to approximately 69.6% and 67.7% of the overall in-flight CO_2_e emissions of CJF & SAF respectively. Two possible ways to decrease this climate impact are by lean combustion engines with less NO_x_ and soot emissions^27^ or by avoiding climate sensitive areas^28–30^. With the new LCCIA WtW CO_2_e emissions values, policy and decision-makers can make informed decisions about the climate impact reduction potential of SAF.

One of the primary reasons why non-CO_2_ effects are currently excluded from the quantification of emissions reduction using CORSIA and EU ETS (RED) is because of the uncertainty associated with their climate impact. We have investigated the impact of a range of uncertainties (see methods) on the equivalent emissions by running a Monte Carlo analysis that generates a whole range of different CO_2_e factors and pump-to-wake results (grey bars in Figs. 2 and 3). To ensure that non-CO_2_ climate impact is fairly and adequately accredited, the CO_2_e factor and PtW results of the Monte Carlo’s 5th percentile can be taken, meaning it is 95% certain that climate impact is at least higher than the accredited value. Moreover, to study the robustness of the model prediction, a sensitivity study has been performed for the NO_x_ RF. By replacing the calculated NO_x_ RF in the AirClim model with the values presented by Lee et al. (2021)^1^, we observed a significant drop in the magnitude of the CO_2_e factors (methodology bar in Supplementary Fig. 5), as the climate-chemistry model that was used in the development of AirClim is at the upper end of the ozone increases per NO_x_ emission of the range given in Lee et al. (2021)^1^. Nevertheless, the variation is within the uncertainty range.

One limitation of this study is that the direct aerosol effects are not considered due to their relatively small values. The absence of sulfur in SAF reduces SO_2_ and volatile H_2_SO_4_ emissions, hence reducing the corresponding cooling effects. Therefore, the CO_2_e ratio for SAF is slightly underestimated in this regard^15^. On the other hand, the cooling effect of sulfur-related emissions might be roughly cancelled out by the direct warming effect of soot emissions (see Supplementary information section 7). The results of the ECLIF-II^17^ and ECLIF-III measurements^31^ indicate that sulfur particles possibly play a role in soot particle activation, as they are thought to increase the soot particle’s hydrophilicity. A higher soot particle activation will lead to a higher number of ice particles with smaller particle diameters, leading to a decreased sedimentation loss of ice particles and therefore longer contrail lifetime. The induced indirect global warming effects due to change in sulfur contents in SAF require thorough analysis.

Furthermore, a recent study^32^ indicates that at sufficiently cold temperatures and low soot emission regime (e.g., lean combustion), background sulfur and organic volatile particles can play critical roles in ice nucleation, hence the contrail radiative forcing. In such situations, the benefit of SAF in reducing the contrail climate effect requires further investigation.

The flight range based CO_2_e ratios shows that the reduction of CO_2_e emissions increases as the flight range increases, this implies that for limited availability of SAF, allocating it to longer flights with higher flight altitudes would allow more climate benefit, as suggested by^33^. Further, the previous study also^33^ showed that the allocation of SAF to night-time flights would have increased climate benefits, as especially the contrails at night have the highest climate impact. If distinct CO_2_e factors for day and night were used, this could lead to substantially lower CO_2_e factors for SAF at night and thereby much higher emissions reductions would be achieved per MJ of SAF. Nevertheless, due to the lifetime of contrails, they are advected into day regions, even if they are produced at night, which complicates, though not rule out, the target SAF use.

Moreover, lean combustion engines emit less NO_x_ and soot and will cause a lower climate impact per unit of fuel used, which means that the CO_2_e factor should also be aircraft type dependent. Even more accurate CO_2_e factors could be made when they would also be weather type dependent as aircraft operators would then be incentivized to allocate their SAF to flights with the highest probability of e.g. contrails cirrus outbreaks. The more factors are considered by the CO_2_e factor, the more accurate the quantification of emissions reductions becomes and the better the incentivization of aircraft operators becomes to use SAF at the right time, in the right aircraft and for the right aircraft trajectory. However, this will also result in more work and cost and possibly require running weather simulations before take-off. Further work should focus on exploring different types of CO_2_e factors, their effect on aircraft operator behavior and the associated cost of making them. In addition, the knowledge and understanding of non-CO_2_ climate impact must be further deepened and uncertainties included in the assessment of mitigation options such as SAF to better understand the impact of those uncertainties on the mitigation potential.

## Methods

The LCCIA method used to derive the WtW values is based on the climate response model, AirClim, which considers a global aviation emission inventory adapted for SAF usage, the growth of aircraft traffic and the relevant background atmospheric species, like CO_2_ and CH_4_. The entire modelling chain is explained in Supplementary Fig. 1. The detail of each element is described in this section.

### Climate assessment

The calculation of global RF and temperature responses resulting from global aviation is performed using AirClim (version 2.0)^34,35^. AirClim is a non-linear climate-chemistry response model to estimate the atmospheric response, radiative forcing, and near-surface temperature changes resulting from the emissions of CO_2_, H_2_O, NO_x_ and contrails. AirClim achieves this by combining emission perturbation data with pre-calculated, altitude- and latitude-dependent atmospheric input data obtained from 85 steady-state simulations performed with the ECHAM4 and E39/CA climate chemistry models. The Supplementary Table 5 shows the RF in 2018 calculated by AirClim model comparing to the literature^1^. To convert the RF to the temperature response, a set of climate sensitivity parameters were taken from the published literature^35^ and are used here in this study. The program does not account for the direct warming effects of soot aerosols nor the direct cooling effects of sulfate aerosols. Indirect aerosol-cloud interactions are omitted from consideration as these are very uncertain, and the climate impacts of CO and unburn hydrogen carbons (UHCs) are also disregarded as they represent a negligible portion of aviation climate impact^1^. A process flow diagram displaying the critical inputs and outputs of AirClim in the context of this research is shown in Supplementary Fig. 1. The three main inputs to generate a baseline 100% CJF scenario are (1) an emissions inventory, (2) a fuel background scenario, and (3) a CO_2_ and CH_4_ background scenario. For the background, we consider an increasing scenario to represent the growth of the air traffic and the involvement of the atmospheric concentrations.

### Emission inventory

The emission inventory used for this research is generated using the Global Aviation Model Emission Inventory (GAMEI) developed in-house^36^. This model generates a 3D grid representing Earth’s atmosphere with a horizontal resolution of 1° by 1° and a vertical resolution of 1000 feet. The fuel consumption, NO_x_ emissions, non-volatile particulate matters nvPM) and flown kilometres by global aviation in 2019 are calculated. To produce this 3D grid, flight departure and destination airports are taken from a flight database of 2019 from flightradar24 which also indicates aircraft type. For each flight, a trajectory is created and segmented into waypoints. At each waypoint, the aircraft’s performance metrics (fuel flow, rate of climb, and velocity) are calculated using BADA (EUROCONTROL) performance data. CO_2_ and H_2_O emissions are calculated by multiplying the used fuel at a certain waypoint with the emission indices of CJF for CO_2_ and H_2_O of 3.16 and 1.25 respectively. NO_x_ emissions for each waypoint are calculated using the Boeing Fuel Flow Method 2 (BFFM2)^37^ and the ICAO Emissions Database (EDB). The result was an emissions inventory with 266.6 Tg fuel, 5.06 Tg NO_x_ and 52.43 × 10^9^ flown km for 2019. The emissions inventory is verified and validated with the emissions inventory from^38^ in Supplementary information (Supplementary Fig. 2 and Supplementary Table 1).

### Fuel background scenario

The fuel background scenarios used in this study are the “Current Technology” (CurTec) and “Business As Usual” (BAU) scenarios taken from^25^ (Supplementary information). These are a top-down series of historic fuel use by global aviation and have been extended from 2100 to 2130. For this extension, an annual Revenue Passenger Kilometer growth of 0.5% per annum has been assumed from 2101 to 2150, and it is assumed that the [#pax/flight] will increase from 85 to 88 for both the CurTec as well as the BAU scenario. Lastly, for CurTec, it is assumed that the fuel efficiency stays 4.67 [kg/km] from 2100 to 2150, whereas for the BAU scenario, this [kg/km] is assumed to decrease with 0.25%/year in 2100 and 0.01%/year in 2150. The Supplementary Fig. 3 shows a good agreement between the temporal evaluation of temperature response using the emission inventory in this work when comparing the literature^25^ under the identify scenario.

### CO_2_ and CH_4_ background scenario

Another important input for AirClim is a CO_2_ and CH_4_ background scenario. For this analysis, the SSP1-2.6 scenario from the IPCC is chosen. The Shared Socioeconomic Pathways (SSP) scenarios are five distinct socio-economic narratives used by the IPCC and in climate change research that predict different global futures based on varying societal developments^39^. SSP1 is the sustainability scenario based on so-called “green road” development. The 2.6 refers to the radiative forcing level, specifically 2.6 W/m^2^ by the year 2100. We have chosen SSP1-2.6 CO_2_ and CH_4_ background scenario, since during the assignment of emissions savings to players in the aviation sector, it must be assumed that all other sectors in all countries are also contributing to emissions savings. In the Supplementary Table 10, the effect of varying the CO_2_ and CH_4_ background scenarios is presented.

### Uncertainty analysis using Monte Carlo

The climate impact of non-CO_2_ effects is associated with a large uncertainty. To account for this, some of the model input parameters, like the CO_2_ and CH_4_ atmospheric residence times, the radiative forcing (RF) strengths and climate sensitivity parameters of all 6 modeled aviation climate agents (CO_2_, CH_4_, H_2_O, O_3_, PMO and contrail cirrus) are prescribed as uncertainty parameters with uniform uncertainty distributions. This gives a total of 11 parameters for which a uniform uncertainty distribution is prescribed and for which 2000 Monte Carlo simulations are done. 2000 simulations were chosen as by then; good convergence (with nominal/default values) was observed.

## Supplementary Information

Below is the link to the electronic supplementary material.

Supplementary Material 1

## Data Availability

All data generated in this study are available in the 4TU.ResearchData repository: https://doi.org/10.4121/eb623e28-e88b-45c4-83bc-928849c57c08.v1.

## References

[1] Lee, D. S. et al. The contribution of global aviation to anthropogenic climate forcing for 2000 to 2018. *Atmos. Environ.* 117834. 10.1016/j.atmosenv.2020.117834 (2021). 10.1016/j.atmosenv.2020.117834 PMC7468346 32895604

[2] Frömming, C. et al. Influence of the actual weather situation on aviation climate effects: the REACT4C climate change functions. *Atmos. Chem. Phys.* **21**, 9151–9172. 10.5194/acp-21-9151-2021 (2021).

[3] Skowron, A., Lee, D. S. & De León, R. R. The assessment of the impact of aviation nox on Ozone and other radiative forcing responses – The importance of representing cruise altitudes accurately. *Atmos. Environ.* **74**, 159–168. 10.1016/j.atmosenv.2013.03.034 (2013).

[4] Stevenson, D. S. et al. Radiative forcing from aircraft nox emissions: mechanisms and seasonal dependence. *J. Geophys. Res. Atmos.* **109** (D17). 10.1029/2004JD004759 (2004).

[5] Myhre, G. et al. *Anthropogenic and Natural Radiative Forcing*. (Cambridge University Press, 2013).

[6] Kärcher, B. Formation and radiative forcing of contrail cirrus. *Nat. Commun.* **9** (1), 1824. 10.1038/s41467-018-04068-0 (2018). 29739923 10.1038/s41467-018-04068-0 PMC5940853

[7] International Civil Aviation Organization (ICAO). *ICAO Guidance on Policy Measures for Sustainable Aviation Fuels (SAF) Development and Deployment*. (version 3, 2024).

[8] European Union (EU), *The EU Emissions Trading System (EU ETS)*. ISBN 978-92-79-62396-7. 10.2834/6083 (2016).

[9] International Civil Aviation Organization (ICAO). *CORSIA Supporting Document: CORSIA Eligible Fuels-Life Cycle Assessment Methodology*. (version 6, 2024).

[10] Yoo, E., Lee, U. & Wang, M. Life-Cycle greenhouse gas emissions of sustainable aviation fuel through a Net-Zero carbon biofuel plant design. *ACS Sustain. Chem. Eng.* **10** (27), 8725–8732. 10.1021/acssuschemeng.2c00977 (2022).

[11] Wang, B., Ting, Z. J. & Zhao, M. Sustainable aviation fuels: key opportunities and challenges in Lowering carbon emissions for aviation industry. *Carbon Capture Sci. Technol.* **13**, 100263. 10.1016/j.ccst.2024.100263 (2024).

[12] Braun, M., Grimme, W. & Oesingmann, K. Pathway to net zero: reviewing sustainable aviation fuels, environmental impacts and pricing. *J. Air Transp. Manage.* **117**, 102580. 10.1016/j.jairtraman.2024.102580 (2024).

[13] Bräuer, T. et al. Reduced ice number concentrations in contrails from low-aromatic biofuel blends. *Atmos. Chem. Phys.* **21** (22), 16817–16826. 10.5194/acp-21-16817-2021 (2021).

[14] Harlass, T. et al. Measurement report: In-flight and ground-based measurements of nitrogen oxide emissions from latest-generation jet engines and 100% sustainable aviation fuel. *Atmos. Chem. Phys.* **24** (20), 11807–11822. 10.5194/acp-24-11807-2024 (2024).

[15] Stratton, R. W. *Life Cycle Assessment of Greenhouse Gas Emissions and non-CO₂ Combustion Effects from Alternative Jet Fuels* (Massachusetts Institute of Technology, Department of Aeronautics and Astronautics, 2010).

[16] Stratton, R. W., Wolfe, P. J. & Hileman, J. I. *Impact of Aviation Non-CO2 Combustion Effects on the Environmental Feasibility of Alternative Jet Fuels*.45. 10736–10743. 2410.1021/es2017522 (Environmental Science & Technology, 2011). 10.1021/es2017522 22106939

[17] Voigt, C. et al. Cleaner burning aviation fuels can reduce contrail cloudiness. *Commun. Earth Environ.* **2** (1), 114. 10.1038/s43247-021-00174-y (2021).

[18] Burkhardt, U., Bock, L. & Bier, A. Mitigating the contrail cirrus climate impact by reducing aircraft soot number emissions. *Npj Clim. Atmospheric Sci.* **1** (1), 37. 10.1038/s41612-018-0046-4 (2018).

[19] Moore, R. H. et al. Influence of jet fuel composition on aircraft engine emissions: A synthesis of aerosol emissions data from the NASA APEX, AAFEX, and ACCESS missions. *Energy Fuels*. **29** (4), 2591–2600. 10.1021/ef502618w (2015).

[20] Moore, R. H. et al. Biofuel blending reduces particle emissions from aircraft engines at cruise conditions. *Nature* **543** (7645), 411–415. 10.1038/nature21420 (2017). 28300096 10.1038/nature21420 PMC8025803

[21] Megill, L., Deck, K. & Grewe, V. Alternative climate metrics to the global warming potential are more suitable for assessing aviation non-CO2 effects. *Commun. Earth Environ.* **5** (1), 249. 10.1038/s43247-024-01423-6 (2024).

[22] Yusaf, T. et al. Sustainable hydrogen energy in aviation – A narrative review. *Int. J. Hydrog. Energy*. **52**, 1026–1045. 10.1016/j.ijhydene.2023.02.086 (2024).

[23] Kurzawska-Pietrowicz, P. Life cycle emission of selected sustainable aviation Fuels – A review. *Transp. Res. Procedia*. **75**, 77–85. 10.1016/j.trpro.2023.12.010 (2023).

[24] Prussi, M. et al. CORSIA: the first internationally adopted approach to calculate life-cycle GHG emissions for aviation fuels. *Renew. Sustain. Energy Rev.* **150**, 111398. 10.1016/j.rser.2021.111398 (2021).

[25] Grewe, V. et al. Evaluating the climate impact of aviation emission scenarios towards the Paris agreement including COVID-19 effects. *Nat. Commun.* **12** (1), 3841. 10.1038/s41467-021-24091-y (2021). 34158484 10.1038/s41467-021-24091-y PMC8219675

[26] Dahlmann, K., Grewe, V., Matthes, S. & Yamashita, H. Climate assessment of single flights: deduction of route specific equivalent CO2 emissions. *Int. J. Sustainable Transp.* **17** (1), 29–40. 10.1080/15568318.2021.1979136 (2023).

[27] Saluja, H. S., Yin, F., Gangoli Rao, A. & Grewe, V. Effects of engine design parameters on the total climate impact: case study based on short-medium range mission. *Aerospace* **10** (12). 10.3390/aerospace10121004 (2023).

[28] Grewe, V. et al. Mitigating the climate impact from aviation: Achievements and results of the DLR WeCare Project. *Aerospace* **4**(3), 34 (2017). 10.3390/aerospace4030034

[29] Teoh, R., Schumann, U., Majumdar, A. & Stettler, M. E. J. *Mitigating the Climate Forcing of Aircraft Contrails by Small-Scale Diversions and Technology Adoption*. 54. 2941–2950. 510.1021/acs.est.9b05608 (Environmental Science & Technology, 2020). 10.1021/acs.est.9b05608 32048502

[30] Yin, F. et al. Predicting the climate impact of aviation for en-route emissions: The algorithmic climate change function submodel ACCF 1.0 of EMAC 2.53. *Geosci. Model Dev*. **16**(11), 3313–3334 (2023). 10.5194/gmd-16-3313-2023

[31] Märkl, R. S. et al. Powering aircraft with 100% sustainable aviation fuel reduces ice crystals in contrails. *Atmos. Chem. Phys.* **24** (6), 3813–3837. 10.5194/acp-24-3813-2024 (2024).

[32] Yu, F., Kärcher, B. & Anderson, B. E. *Revisiting Contrail Ice Formation: Impact of Primary Soot Particle Sizes and Contribution of Volatile Particles*. 58. 17650–17660. 4010.1021/acs.est.4c04340 (Environmental Science & Technology, 2024). 10.1021/acs.est.4c04340 PMC11465739 39323293

[33] Teoh, R. et al. *Targeted Use of Sustainable Aviation Fuel to Maximize Climate Benefits*. 56. 17246–17255. 2310.1021/acs.est.2c05781 (Environmental Science & Technology, 2022). 10.1021/acs.est.2c05781 PMC9730838 36394538

[34] Grewe, V. & Stenke, A. AirClim: an efficient tool for climate evaluation of aircraft technology. *Atmos. Chem. Phys.* **8** (16), 4621–4639. 10.5194/acp-8-4621-2008 (2008).

[35] Dahlmann, K., Grewe, V., Frömming, C. & Burkhardt, U. Can we reliably assess climate mitigation options for air traffic scenarios despite large uncertainties in atmospheric processes? *Transp. Res. Part. D: Transp. Environ.* **46**, 40–55. 10.1016/j.trd.2016.03.006 (2016).

[36] Kroon, R. *Aviation Emission Inventory: A Contemporized Bottom-up Emission Inventory for the Year 2019* (Delft University of Technology, 2022).

[37] DuBois, D. & Paynter, G. C. Fuel flow Method2 for estimating aircraft emissions. In *SAE Technical Paper 2006-01-1987* (2006). 10.4271/2006-01-1987

[38] Teoh, R., Engberg, Z., Shapiro, M., Dray, L. & Stettler, M. E. J. The high-resolution global aviation emissions inventory based on ADS-B (GAIA) for 2019–2021. *Atmos. Chem. Phys.* **24** (1), 725–744. 10.5194/acp-24-725-2024 (2024).

[39] Riahi, K. et al. The shared socioeconomic pathways and their energy, land use, and greenhouse gas emissions implications: an overview. *Glob. Environ. Change*. **42**, 153–168. 10.1016/j.gloenvcha.2016.05.009 (2017).

