# Supplementary material for: A comprehensive well-to-wake climate impact assessment of sustainable aviation fuel
Source: Sci Rep. 2025 Aug 30;15:31966. doi: 10.1038/s41598-025-13445-x (PMC12398561; doi:10.1038/s41598-025-13445-x)
Supplement: Supplementary file 1 — Supplementary Material 1 [file 41598_2025_13445_MOESM1_ESM.pdf]

Supplementary information for: *A comprehensive well-to-wake climate impact assessment of Sustainable Aviation Fuel*

Luc Boerboom[1], Arvind Gagnoli Rao[1], Volker Grewe[1,2], Feijia Yin[1,*]

1. Falcuty of Aerospace Engineering, Delft University of Technology, The Netherlands
2. Deutsches Zentrum fur Luft- und Raumfahrt, Institut fur Physik der Atmosphare, Oberpfaffenhofen, Germany.

*Corresponding author: Email: f.yin@tudelft.nl

SI 1: Model Chain Overview

Figure S1 shows the modeling chain used for obtaining CO_2e values for the individual effect of contrails, NO_x, water vapor and consequently pump-to-wake (PtW) and Well-to-Wake (WtW) "results". The state-of-the-art climate model, AirClim [1, 2], is used as a tool for making CO_2e ratios and is entirely decoupled from the well-to-pump (WtP) analysis. Well-to-pump values can be taken from the lifecycle analyses (LCAs) of CORSIA.

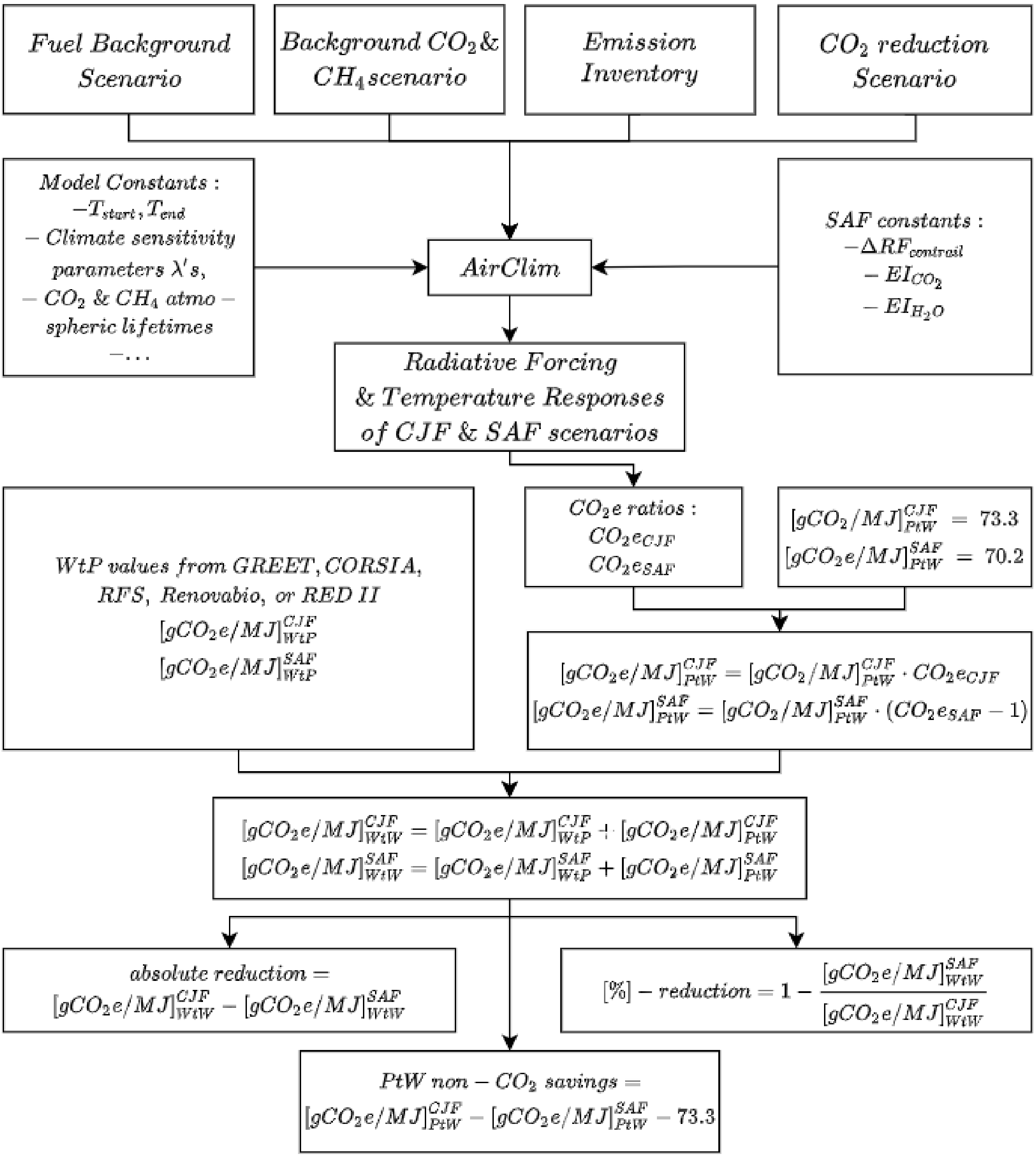

Supplementary Figure 1: Method overview

In this research, we use Average Temperature Response (ATR) and Global Warming Potential (GWP) as climate metrics. The changes in contrail RF by using SAF is derived using the methods described in the section SI 3 of this supplement.

SI 2: Comparison of Emissions Inventory to Literature

The emissions inventory is generated using an in-house model developed by Kroon (2022) [3]. The emissions inventory is a 3D grid that represents the Earth's atmosphere with a resolution 1 degree x 1 degree x 1000 ft (latitude x longitude x altitude) and thus consists of 360 x 180 x 50 = 3.24 million cells. Each cell contains the fuel consumed, NO_x emitted, and km traveled in that cell for the entire year of 2019. The model output is compared to the results from Teoh, Engberg [4], which is a global emissions inventory based on Automatic Dependent Surveillance–Broadcast (ADS-B) telemetry data – and the aircraft performance model (BADA3 performance data) and emission model (BFFM2 + ICAO EDB). Figure SI 2 shows the vertical profile of the fuel burn calculated from both studies.

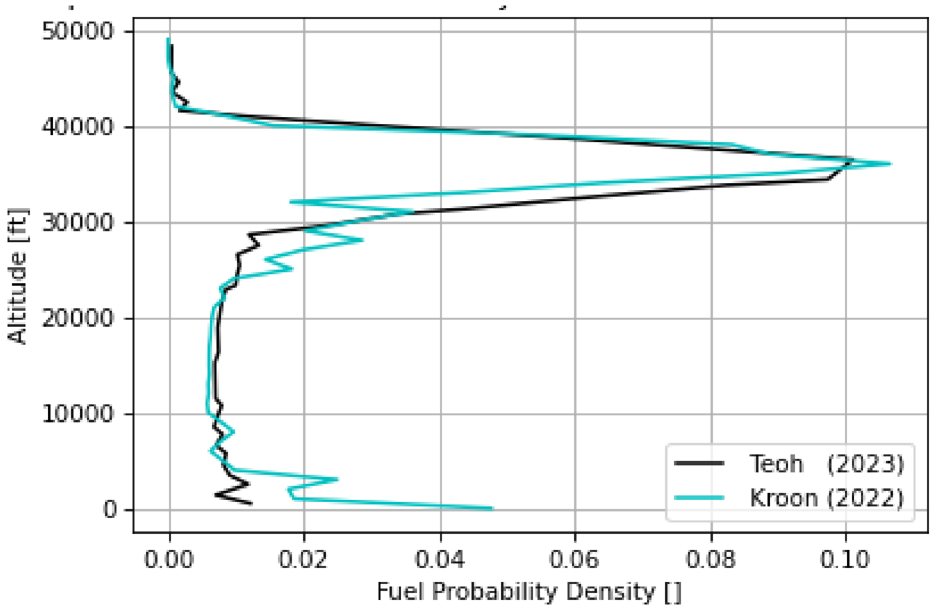

Supplementary Figure 2: Fuel-Altitude Probability Density Function of the emission inventory from Kroon (2022) corresponding to the total values given in table SI 2. Both emission inventories have the same altitude resolution of 1000 ft. The values from figure S13(c) of Teoh, Engberg [4] are taken for comparison.

The discrepancy at low altitude can be explained by the fact that Teoh et al. (2024) [4] do not include emissions made at ground level, whereas Kroon (2022) [3] included those values (also indicated in Supplementary Table 1). In this analysis, we consider ground emissions for both SAF and CJF (scenarios 1 and 2). The discrepancies at the 25-30 kft altitude can be explained by the fact that Teoh et al. (2024) [4] uses actual ADS-B flight data and Kroon (2022) [3] uses a model to determine the rate of climb and performs a climbing step once a certain threshold is exceeded. The total fuel consumed, NO_x emitted, and flown km are given in Supplementary Table 1. Only the Fuel, NO_x and flown kilometers (in $[bkm] = 109km$) are compared since these are the inputs for AirClim and will have an influence on the result.

Inventory	Scope	Fuel [Tg]	NO_x [Tg]	[bkm] flown	EI NO_x [g/kg]	[kg/km]
Kroon (2022) [3]	including ground	266.6	5.1	52.4	19.0	5.1
	excluding ground	252.1	4.9	52.4	19.4	4.8
Teoh et al. (2024) [4]	incl. piston turboprop	283	4.49	61.0	15.9	4.6

	excl. piston turboprop*	279.07	4.43	59.0	15.9	4.7
Lee et al. (2021) [5]	Civil Aviation (2018)	310.8	4.7	61.3	15.14	5.1

Supplementary Table 1: Overview comparison of Fuel, NO_x and flown (10^9) [km] in 2019 from different emission inventory calculations [3, 4] and the values used by Lee et al. (2021) [5].

Teoh et al. (2024) [4] includes emissions from piston and turboprop aircraft and for a proper comparison between the two inventories, the fuel consumed, NO_x emitted, and km flown by these aircraft have to be subtracted. Although piston and turboprop aircraft represent 17.4% of the total flights, their contribution to total fuel consumption, NO_x emission and flown km is relatively small. In addition, the altitude at which they fly is lower compared to commercial jet aircraft, which lowers their contribution to climate impact, so the fact that they are not included in the inventory from Kroon (2022) [3] used in this research is not such a big issue.

Teoh et al (2024) [4] analyze 40.2 million flights in 2019, of which roughly 33.4 million (83%) are jet aircraft. Kroon (2022) [3] on the other hand analyzes 555,209 flights in the representative week, giving 29 million flights[1] for the whole of 2019, which is ± 13% lower than Teoh et al. (2024) [4]. This explains why the flown km of all inventories is 11% lower and why the fuel consumed is 9.7% lower. The fuel efficiencies on the other hand are very similar. The most important difference is the $EINO_x$. The emissions inventory from [6] shown an EI_{NOx} of 15.56 and Lee et al (2021) uses an EI_{NOx} of 15.14 to quantify the radiative forcing. On the other hand, a recent emissions inventory from [7] estimated a higher EI_{NOx} of 18.9.

SI 3: Reduction in Contrail RF due to SAF Blending

SAF has a lower aromatic content and therefore reduces the nvPM emissions [8]. This in turn leads to a lower number of ice crystals n_{ice} with bigger diameters D_{ice} in the contrail plume and these larger ice particles tend to sediment faster and reach ice subsaturated layers of the atmosphere earlier, allowing the ice crystals to sublimate more easily. This reduces contrail lifetime and coverage and thus leads to a lower $RF_{contrail}$. To quantify this reduction in $RF_{contrail}$ (or $\Delta RF_{contrail}$) due to SAF blending, firstly the reduction in $EI_{n,nvPM}$ (or $\Delta EI_{n,nvPM}$) has to be quantified since the soot emissions are the most dominant factor contributing to n_{ice} (in the soot rich regime) [9] [10]. To this end, the methodology proposed in [11] for quantifying nvPM number emission index as a function of a fuel hydrogen content shown in Eqn. (1) is used:

$$\Delta EI_{n,nvPM}\ [\%] = \begin{cases} (a_0\ +\ a_1\ F\ )\ x\ \Delta H, & when\ \Delta H\ \leq\ 0.5\% \\ (a_0 + a_1\ F\ )\ x\ \Delta H\ x\ e^{0.5x(0.5-\Delta H)}, & when\ \Delta H\ >\ 0.5\% \end{cases} \quad (1)$$

Where $\hat{F}$ is the corrected thrust setting. α_0 and α_1 equal -114.21 & 1.06 respectively. The ΔH is the increase of the SAF's hydrogen content [mol%] w.r.t. a CJF baseline. This method for quantifying soot emissions reductions is most applicable to Rich burn Quick Quench Lean burn (RQL) engines, and in reality, the actual $\Delta EI_{n,nvPM}$ due to SAF blending will be different for every engine. However, as most aircraft in service today use RQL technology, and the share of lean burning engines like the Leap-1A and GEnx is relatively low, this relation is used in this research.

Using Eqn. (2) from [12] that is based on simulations from [13], the reduction in $RF_{contrail}$ can be quantified as a function of $\Delta EI_{n,nvPM}$. It is assumed that n_{ice} reduces linearly with nvPM emissions, which holds for the soot-rich regime, where soot particles regulate ice crystal formation [9] [10].

[1] The exact calculation is done as follows: 555,209 flights/week x 365/7 days / week x 808,637/806,631 = 29 million . The latter term is a correction factor used to account for the fact that the representative week has a lower mean level of flights.

$$\Delta RF_{contrail}\ [\%] = \frac{\arctan{(1 + \Delta n_{ice})}^{0.74}}{\arctan{(1.9)}} = \frac{\arctan{(1 + \Delta EI_{n,nvPM})}^{0.74}}{\arctan{(1.9)}} \qquad (2)$$

Supplementary Table 3 shows how the SAF blend level can be related to $\Delta RF_{contrail}$ with a thrust level $\hat{F}$ of 60% being considered. For the $\Delta EI_{n,nvPM}$, both the reductions due to SAF blending (Eqn.1) as well as due to the lower fuel flow of SAF are taken into account. The reduction in $EI_{n,nvPM}$ resulting from the lower fuel flow of SAF is calculated by multiplying the blend level with (1 − 43.04/44.2)[2]. Following this analogy, a SAF blend level of 100% and a thrust level $\hat{F}$ of 60% would mean that contrail RF is decreased by 20.9%.

Blend level	H_{fuel} [%]	ΔH [%]	$\Delta EI_{n,nvPM}$ [%]		$\Delta RF_{contrail}$
			eq. 3.1	fuel	
0%	13.8	0	0	0	0%
1%	13.815	0.015	-0.76	-0.03	-0.22%
10%	13.95	0.15	-7.59	-0.26	-2.34%
20%	14.1	0.3	-15.18	-0.52	-4.96%
30%	14.25	0.45	-22.77	-0.79	-7.95%
40%	14.4	0.6	-28.89	-1.05	-10.68%
50%	14.55	0.75	-33.50	-1.31	-12.99%
60%	14.7	0.9	-37.29	-1.57	-15.08%
70%	14.85	1.05	-40.36	-1.84	-16.93%
80%	15	1.2	-42.80	-2.10	-18.52%
90%	15.15	1.35	-44.67	-2.36	-19.84%
100%	15.3	1.5	-46.04	-2.62	-20.9%

Supplementary Table 2: $\Delta EI_{n,nvPM}$ & $\Delta RF_{contrail}$ as function of SAF blend level assuming $\hat{F}$ = 60%

	$\Delta EI_{n,nvPM}$ [% difference]			$\Delta RF_{contrail}$
$\hat{F}$	eq. 1	Fuel Consumption	Total	
50	-55.7	-2.62	-58.31	-28.0%
60	-46.0	-2.62	-48.67	-20.9%
70	-36.4	-2.62	-39.03	-15.2%
80	-26.8	-2.62	-29.38	-10.4%
52.5	-53.3	-2.62	-55.90	-26.0%

Supplementary Table 3: $\Delta EI_{n,nvPM}$ & $\Delta RF_{contrail}$ as function of thrust level $\hat{F}$ for 100% SAF blend level (ΔH = 1.5%)

When comparing the $\Delta RF_{contrail}$ of 20.9% to other values in literature given in Supplementary Table 4, it is noted that there is a lot of debate about how much $\Delta RF_{contrail}$ can be achieved with lower n_{ice}. Firstly, for simulations looking at the potential present-day $\Delta RF_{contrail}$ due to Δn_{ice} of -50% to -56%, the values range from -44% to -20%. The values from [14] are left out of the discussion, because according to Teoh et al (2022) [11], the ΔRF from Caiazzo et al. (2017) [14] is the change in mean contrail net RF[3] and not the annual mean net RF[4]. The trend observed is that the Contrail Cirrus

[2] These are the energy densities of CJF and SAF.

[3] which looks at change in contrail RF locally & increases because of the reduced albedo (Twoomey) effect

[4] which takes into account the shorter lifetime of contrails

Prediction model (CoCiP) generally predicts more $\Delta RF_{contrail}$. On top of this, the ECLIF-III in-flight experimental measurements suggests that not only SAF lowers $\Delta EI_{n,nvPM}$, but also its lower EI_{SO2} could lead to a reduction in n_{ice}, which would mean that the $RF_{contrail}$ of -20.9% is too pessimistic.

Literature	Domain (year) and model	[%]-changes		
		$\Delta EI_{n,nvPM}$	Δn_{ice}	$\Delta RF_{contrail}$
Burkhardt, 2018	Global (2006) ECHAM-CCMod	-	-50%	-20%
		-	-80%	-50%
Bier & Burkhardt, 2022		-	-80%	-41%
Bock & Burkardt, 2019	Global (2050) ECHAM-CCMod	-	-50%	-14%
Teoh et al., 2022	North Atlantic (2019) CoCiP	-52%	-55%	-44%
Caiazzo, 2017	US (2017) CERM	-75%	-75%	(-4,18)%
Märkl, 2024	Global (2018) CoCiP + ECHAM-CCMod	-32%	-56%	-26%
Schumann, 2013	Global CoCiP	-50%	-	-39%

Supplementary Table 4: Overview of decrease in RFcontrail due to a decrease in #ice from previous studies [11].

On the other hand, Bock et al. [15] found a $\Delta RF_{contrail}$ of -14% in 2050 for a Δn_{ice} of -50%, whereas in Burkhardt et al. (2018), this same model with the same Δn_{ice} is used in the year 2006 and there, a $\Delta RF_{contrail}$ of -20% is predicted. The reason for this is that for higher air traffic scenarios, reductions in n_{ice} lead to lower $\Delta RF_{contrail}$. This means that SAF blending in the future would thus make less sense and give a lower $\Delta RF_{contrail}$. Taking all these effects into account, a $\Delta RF_{contrail}$ of -20.9% due to a $\Delta EI_{n,nvPM}$ of roughly -50% is considered to be a reasonable assumption.

SI 4 : Comparison of Climate Model Results to Literature

Using the inputs of AirClim shown in Supplementary Figure 1, a radiative forcing and a temperature response for a CJF and a SAF scenario can be generated with AirClim (see figure 1 in main). For the baseline CJF scenario (case (1) in main), the RF responses of all species are compared to values from of Lee et al. (2021) [5] for the year 2018 in Supplementary Table 5. This table also compares contrail RF in 2006 and 2050 with Bock & Burkhardt (2019) [15] for the years 2006 and 2050. In Supplementary Figure 3, the temperature change response generated with AirClim is compared to the "Business as Usual (BAU)" temperature change scenario from Grewe et al. (2021) [12], which has also been generated with AirClim using the same background fuel development scenario (BAU) and a very similar CO_2 and CH_4 background scenario (SSP1-2.6).

Climate Agent	RF [mW/m^2] in 2018	
	Lee et al., 2021	Kroon, 2022
Contrails	111.4 (33,189)	76.26
CO_2	34.3 (31, 38)	21.8
O_3	36 (23, 56)	83.25
PMO	-9 (-17, -6.3)	-6.84
CH_4	-17.9 (-34, -13)	-23.75

SWV	-2.7 (-5.0, -1.9)	n.a.
net NO_x	8.2 (-4.8, 16)	52.65
H_2O	2 (0.8, 3.2)	3.82
Soot aerosol	0.94 (0.1, 4.0)	n.a.
Sulfur aerosol	-7.4 (-19, -2.6)	n.a.
Total	149.1	154.9
Total of contrail, CO_2, NO_x, H_2O	158.3	154.9
	Bock and Burkhardt, 2019	Kroon, 2022
RF contrail 2006	49	50.7
RF contrail 2050	160	111.4

Supplementary Table 5: Radiative Forcing [mW/m^2] in 2018 generated by AirClim compared to literature [5] [15].

Firstly, RF_{CO2} in 2018 is reasonably estimated, considering that Lee et al. (2021) [5] use 310.8 Tg_{fuel} in 2018 (which is 95% of IEA estimate of 327 Tg_{fuel} for total aviation), whereas Kroon (2022) [3] outputs 266 Tg_{fuel} for 2019, which means that the discrepancy can be attributed to the incompleteness of the flightradar24 flight dataset (information model) which serves as the basis for the emissions inventory.

Secondly, $RF_{contrail}$ is also in reasonable agreement with Lee et al. (2021) [5], bearing in mind that Lee et al., (2021) [5] uses 61.3 [bkm] in 2018 in contrast to 52.43 [bkm] in Kroon (2022) [3] for 2019 (AirClim calculates $RF_{contrail}$ as [$mW/m2/km$]·[km]). Moreover, in Märkl et al. (2024) [16], besides discussing the ECLIF-III measurement results, the global $RF_{contrail}$ is also estimated for a 100% Jet A-1 scenario and is found to be 72 mW/m^2 for 2018, using ECHAM5-CCMod and an AEDT inventory. For 2006, [15] calculate 49 mW/m^2, which agrees well with the 50.7 mW/m^2. In Bier and Burkhardt [17], this contrail RF has been recalculated (updated) to be 44 mW/m^2 instead of 49 though, but this is still in reasonable agreement. The reason why Bock & Burkhardt (2019) have a higher $RF_{contrail}$ in 2050 is that they have 154 billion traveled km, whereas for Kroon (2022), the traveled km in 2050 for BAU equals 121.4 [bkm]. In addition, Bock & Burkhardt (2019) incorporate the effect of higher-flying aircraft in the future. The authors also incorporate the effect of a changing background climate (more humidity and global temperature), but this effect is found to be very small.

The NO_x-induced RF_{O3} is very different from the Lee et al. (2021) estimate and far outside of its 5-95% confidence interval. One might be tended to say that this is caused by the higher EI_{NOx} of ± 19 [g/kg] compared to 15.14 used in Lee et al. (2021). However, this is not the cause, because Lee et al. (2021) multiply this 15.14 with 310 Tg_{fuel}, resulting in 4.7 Tg_{NOx} for 2018, which is quite similar to the 5.06 Tg_{NOx} emitted in the Kroon (2022) inventory in 2019 shown in table 3.1. This similar total NO_x emission also explains why the RF_{PMO} & RF_{CH4} show a more reasonable agreement. It has been tested if the RF_{O3} could be reduced by artificially making the NO_x of the inventories lower, but this gave only a slight reduction in RF_{O3}. This means that neither the NO_x magnitude, nor the altitude at which the NO_x is emitted have a big impact in reducing RF_{O3}, which suggests that the reason why the RF_{O3} is so high is due to the employed methodology. For this reason, the NO_x RF calculation methodology is varied as a part of the sensitivity study in SI 8.

RF_{H2O} is higher than the estimate from Lee et al. (2021), but within the uncertainty range. If more fuel would be used in the inventory, it would probably be outside the uncertainty range again. The high RF_{H2O} can again be explained by the methodology used in AirClim to calculate RF_{H2O}.

Overall, when ignoring the contributions of soot, sulfates and $NO_x - SWV$, the total RF's are quite similar, which essentially results from the contrail underestimation and the NO_x-O_3 overestimation. It

should also be kept in mind that the values from Lee et al. (2021) are multi-model means and are not perfect either- especially not for contrails.

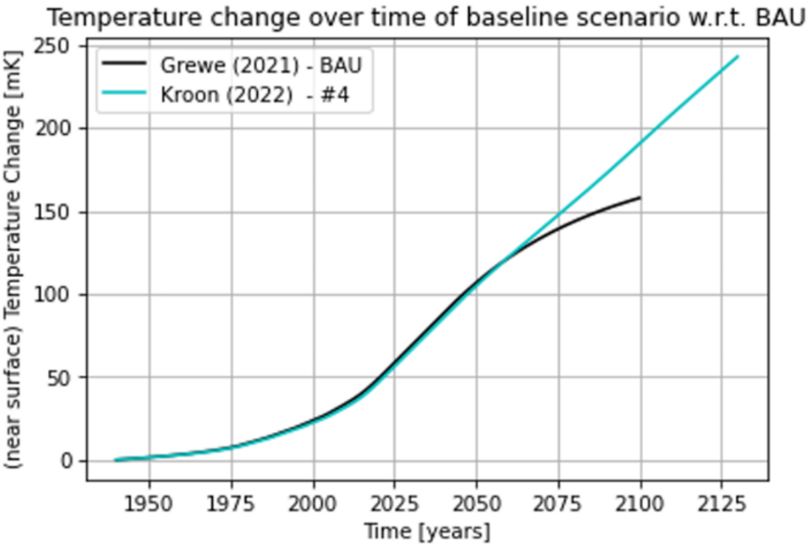

Supplementary Figure 3: Comparison of temperature change response generated with AirClim for scenario (1) - using baseline settings - to Business As Usual (BAU) temperature development from Grewe et al. (2021), which was also generated with AirClim.

Supplementary Figure 3 shows very good agreement between the two temperature change responses from 1940-2060. The diversion between the lines after around 2060 is due to the difference in emissions inventory and more specifically, the high EI_{NOx} of Kroon (2022) inventory w.r.t. the WeCare inventory (with EI_{NOx} = 14.3) used for the black BAU line. Also, the fact that the (black) BAU line is made with a slightly different background CO_2 and CH_4 background concentrations (RCP2.6 instead of SSP1-2.6) plays a role.

SI 5: CO_2e ratio based on ATR in tabulated form

The CO_2e ratios in Supplementary Table 6 correspond to those in Figure 2 of this study. 2000 Monte Carlo simulations have been done and for each simulations, the CO_2e ratios for all species and PtW results ([gCO_2e/MJ]$_{PtW}$) for CJF and SAF are calculated. Based on this, the WtW absolute and [%]-emissions reductions of a perfect SAF with 0 WtP emissions are also calculated. As the number of simulations increases, the average values of the Monte Carlo analysis converge more towards the default settings. 2000 simulations are chosen, since for this #simulations, decent convergence is observed.

			**Monte Carlo Results - ATR20/50/100**			**Default Settings**
			5th percentile	average	95th percentile	
**ATR100**	**CJF**	CO_2e CO_2	1	1	1	1
		CO_2e H_2O	0.06	0.11	0.19	0.11
		CO_2e contrail	0.43	0.77	1.13	0.76
		CO_2e NO_x	0.65	1.4	2.32	1.42
		CO_2e total	2.42	3.29	4.29	3.29
		[gCO_2e/MJ]$_{PtW}$	177.2	240.8	314.5	241.5
	**SAF**	CO_2e CO_2	0	0	0	0
		CO_2e H_2O	0.06	0.13	0.21	0.13

		CO_2e contrail	0.31	0.56	0.83	0.55
		CO_2e NO_x	0.66	1.42	2.36	1.42
		CO_2e total (incl. CO_2)	2.29	3.11	4.1	3.10
		$[gCO_2e/MJ]_{PtW}$	90.3	148.1	217.7	147.7
	$[gCO2e/MJ]_{WtW}$ [%-reduction]		33.8%	43.2%	53.3%	42.6%
	$[gCO2e/MJ]_{WtW}$ [absolute-reduction]		99.3	108.4	118.5	109.5
	Additional non-CO2 savings $[gCO2e/MJ]_{PtW}$		10.3	19.4	29.5	20.5
**ATR50**	**CJF**	CO_2e CO_2	1	1	1	1
		CO_2e H_2O	0.08	0.11	0.27	0.16
		CO_2e contrail	0.66	1.19	1.75	1.18
		CO_2e NO_x	0.97	2.09	3.45	2.11
		CO_2e total	3.15	4.44	5.94	4.45
		$[gCO_2e/MJ]_{PtW}$	230.8	325.5	435.5	326.5
	**SAF**	CO_2e CO_2	0	0	0	0
		CO_2e H_2O	0.09	0.19	0.31	0.19
		CO_2e contrail	0.47	0.84	1.24	0.83
		CO_2e NO_x	0.98	2.1	3.48	2.1
		CO_2e total (incl. CO_2)	2.92	4.13	5.6	4.12
		$[gCO_2e/MJ]_{PtW}$	134.8	219.8	322.7	219.2
	$[gCO2e/MJ]_{WtW}$ [%-reduction]		28.0%	36.5%	45.8%	35.90%
	$[gCO2e/MJ]_{WtW}$ [absolute-reduction]		106.6	121.4	138.0	122.96
	Additional non-CO2 savings $[gCO2e/MJ]_{PtW}$		17.6	32.4	49.0	34
**ATR20**	**CJF**	CO_2e CO_2	1	1	1	1
		CO_2e H_2O	0.1	0.11	0.34	0.21
		CO_2e contrail	0.89	1.6	2.36	1.59
		CO_2e NO_x	1.44	2.95	4.76	2.98
		CO_2e total	4.02	5.76	7.77	5.78
		$[gCO_2e/MJ]_{PtW}$	294.7	422.3	569.8	423.6
	**SAF**	CO_2e CO_2	0	0	0	0
		CO_2e H_2O	0.12	0.24	0.39	0.24
		CO_2e contrail	0.6	1.09	1.62	1.08
		CO_2e NO_x	1.45	2.93	4.74	2.93
		CO_2e total (incl. CO_2)	3.65	5.26	7.21	5.25
		$[gCO_2e/MJ]_{PtW}$	186.4	299.2	435.6	298.4
	$[gCO2e/MJ]_{WtW}$ [%-reduction]		24.8%	32.5%	41.0%	32.10%
	$[gCO2e/MJ]_{WtW}$ [absolute-reduction]		116.7	138.8	163.7	140.95
	Additional non-CO2 savings $[gCO2e/MJ]_{PtW}$		27.7	49.8	74.7	51.9

Supplementary Table 6: Results of Monte Carlo analysis with 2000 simulations. CO_2e ratios and PtW results based on Average Temperature Response climate metric with 100-year, 50-year and 20-year time horizon.

SI 6: CO_2e ratios based on Global Warming Potential

While Figure 2 of this study is based on ATR CO_2e results, a similar analysis can be done for the GWP CO_2e ratios with 20-, 50- and 100-year timeframes by looking at the radiative forcing responses from Figure 1 in the main text and using Eqn. $eq. SI$ 6.1, where the subscript i denotes the individual climate specie of NO_x, H_2O, contrail and CO_2.

$$CO_{2e,all,GWP_H} = \frac{\sum_i \int_{2019}^{2019+H} RF_i(t) - RF_i(2019)\, dt}{\int_{2019}^{2019+H} RF_{CO_2}(t) - RF_{CO_2}(2019)\, dt} \qquad (eq.\ SI\ 6.1)$$

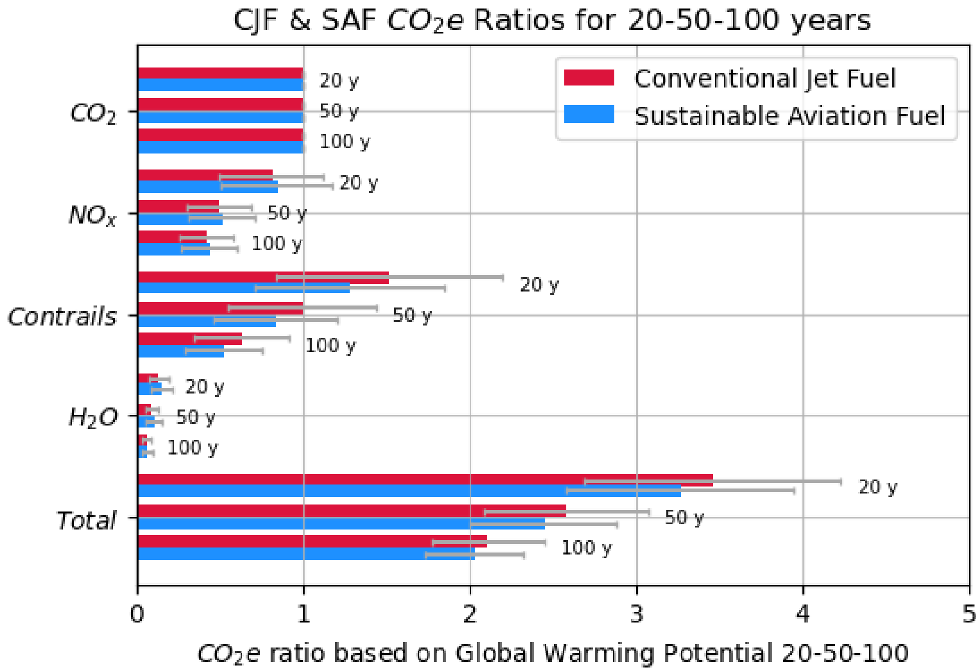

Supplementary Figure 4: CO_2e ratios of CO_2, NO_x, contrails, H_2O and total based on the Average Temperature Response over 20, 50 & 100 years. Colored bars represent average and uncertainty bars denote 5th & 95th percentiles coming from a Monte Carlo analysis with 2000 simulations. The CO_2e ratios for 100-year time horizon are also given in tabulated form in Supplementary Table 7.

These GWP CO_2e ratios are lower than the ATR CO_2e ratios from Figure 2 because non-CO_2 effects have higher efficacies and climate sensitivity parameters than CO_2. The climate sensitivity parameter λ_i indicates how temperature change is caused by 1 unit of radiative forcing of a species *i* and the efficacy r_i relates the climate sensitivity parameter of 1 species *i* to that of CO_2. Also, the uncertainty ranges of the GWP CO_2e ratios are lower than those based on ATR, since there is uncertainty associated with the climate sensitivity parameters. The λ_i's and r_i's used for the default simulation are taken from Ponater et al. (2006) and the uncertainty distributions for the λ_i's are taken from Dahlmann et al. (2016).

			Monte Carlo Results - GWP20/50/100			Default Settings
			5th percentile	average	95th percentile	
**GWP100**	**CJF**	CO_2e CO_2	1	1	1	1
		CO_2e H_2O	0.03	0.06	0.09	0.06
		CO_2e contrail	0.35	0.63	0.91	0.62
		CO_2e NO_x	0.26	0.42	0.58	0.43
		CO_2e total	1.77	2.11	2.45	2.11
		[gCO_2e/MJ]$_{PtW}$	130.0	154.7	179.4	154.6
	**SAF**	CO_2e CO_2	0	0	0	0
		CO_2e H_2O	0.04	0.07	0.1	0.07
		CO_2e contrail	0.29	0.52	0.76	0.52
		CO_2e NO_x	0.26	0.44	0.6	0.44
		CO_2e total (incl. CO_2)	1.73	2.03	2.33	2.02
		[gCO_2e/MJ]$_{PtW}$	50.9	72.1	93.1	71.5
	[gCO2e/MJ]$_{WtW}$ [%-reduction]		52.2%	58.1%	65.1%	58.0%
	[gCO2e/MJ]$_{WtW}$ [absolute-reduction]		93.9	98.4	103.1	98.8
	Additional non-CO2 savings [gCO2e/MJ]$_{PtW}$		4.9	9.4	14.1	9.8
**GWP50**	**CJF**	CO_2e CO_2	1	1	1	1
		CO_2e H_2O	0.05	0.09	0.13	0.09
		CO_2e contrail	0.55	1	1.44	0.99
		CO_2e NO_x	0.3	0.5	0.68	0.50
		CO_2e total	2.08	2.58	3.08	2.58
		[gCO_2e/MJ]$_{PtW}$	152.7	189.3	225.5	189
	**SAF**	CO_2e CO_2	0	0	0	0
		CO_2e H_2O	0.06	0.1	0.15	0.1
		CO_2e contrail	0.46	0.83	1.2	0.82
		CO_2e NO_x	0.31	0.51	0.71	0.51
		CO_2e total (incl. CO_2)	2	2.45	2.88	2.44
		[gCO_2e/MJ]$_{PtW}$	70.5	101.8	132.3	100.8
	[gCO2e/MJ]$_{WtW}$ [%-reduction]		45.1%	50.8%	57.9%	50.70%
	[gCO2e/MJ]$_{WtW}$ [absolute-reduction]		96.4	103.2	110.3	103.9
	Additional non-CO2 savings [gCO2e/MJ]$_{PtW}$		7.4	14.2	21.3	14.9
**GWP20**	**CJF**	CO_2e CO_2	1	1	1	1
		CO_2e H_2O	0.07	0.13	0.19	0.13
		CO_2e contrail	0.84	1.52	2.2	1.51
		CO_2e NO_x	0.49	0.81	1.12	0.82
		CO_2e total	2.69	3.46	4.23	3.46
		[gCO_2e/MJ]$_{PtW}$	197.2	253.8	310.0	253.4
	**SAF**	CO_2e CO_2	0	0	0	0
		CO_2e H_2O	0.08	0.15	0.22	0.15
		CO_2e contrail	0.7	1.28	1.84	1.26
		CO_2e NO_x	0.51	0.85	1.17	0.84
		CO_2e total (incl. CO_2)	2.58	3.27	3.95	3.25
		[gCO_2e/MJ]$_{PtW}$	111.1	159.5	206.9	158.1
	[gCO2e/MJ]$_{WtW}$ [%-reduction]		36.2%	41.2%	47.7%	41.30%
	[gCO2e/MJ]$_{WtW}$ [absolute-reduction]		99.8	110.0	120.7	111.1
	non-CO_2 savings [gCO2e/MJ]$_{PtW}$		10.8	21.0	31.7	22.1

Supplementary Table 7: Results of Monte Carlo analysis with 2000 simulations. CO_2e ratios and PtW results based on Average Temperature Response climate metric with 100-year, 50-year and 20-year time horizon.

SI 7: Verification with respect to literature

The CO_{2e} values calculated in this study are compared to Stratton (2010) [18] in Supplementary Table 8 for the GWP100 and to those of Dahlmann, Grewe [19] in Supplementary Table 8 for the ATR100

		This research	Stratton, 2010
**CJF**	CO_2e CO_2	1	1
	CO_2e H_2O	0.06	0.18
	CO_2e Contrail	0.62	0.93
	CO_2e NO_x	0.43	0.05
	CO_2e Sulfates	n.a.	-0.28
	CO_2e Soot	n.a.	0.18
	CO_2e Total	2.11	2.06
	$[gCO_2e/MJ]_{PtW}$	154.64	151.0
**SAF**	CO_2e CO_2	0	0
	CO_2e H_2O	0.07	0.2
	CO_2e Contrail	0.52	0.96
	CO_2e NO_x	0.44	0.048
	CO_2e Sulfates	n.a.	0
	CO_2e Soot	n.a.	0.015
	CO_2e Total (incl. CO_2)	2.02	2.223
	$[gCO_2e/MJ]_{PtW}$	71.50	85.9
Reduction of $[gCO_2e/MJ]_{WtW}$ [%]	58%	48.5%	
Reduction of $[gCO_2e/MJ]_{WtW}$	98.83	80.8	
non-CO_2 savings $[gCO_2e/MJ]_{PtW}$	9.83	-8.2	

Supplementary Table 8: Comparison of GWP100 CO_2e ratios & $[gCO_2e/MJ]_{PtW}$ to [18]. Also, WtW [%]-$reductions$ & $[absolute\ reductions]$ for a SAF with $[gCO_2e/MJ]_{WtP}$ = 0 w.r.t. CJF with $[gCO_2e/MJ]_{WtP}$ = 15.7 are compared.

In Supplementary Table 8, the two studies have used different climate models, therefore, we generally expect discrepancies in the absolute values. Stratton [18] calculates a much higher H_2O effect and a much lower NO_x CO_2e. Also, Stratton considers the cooling effect of sulfates and the (direct) warming effect of soot particles. However, their influence on the total CO_2e ratio is quite small since their effects nearly cancel out. When switching from CJF to SAF, a large discrepancy is observed for the contrail CO_{2e} because Stratton [18] did not account for the reduced radiative forcing from contrails when burning SAF.

The CJF ATR100 based CO_2e and the PtW CO_{2e} in g/MJ results are evaluated against the outcome of [19]. The authors investigated the carbon footprint of single flights of the A330-200 using ATR100 based CO_2e. Supplementary Table 9 shows that Dahlmann (2023) has higher values for $CO_2e\ H_2O$ and $CO_2e\ contrail$, but lower values for $CO_{2e}\ NOx$. This can be explained by the fact that A330-200 are long-haul aircraft flying at the higher (especially at the end of the flights), which makes their H_2O & contrail climate impact higher. The reason why the NOx climate impact is lower for Dahlmann, Grewe [19] is because of the higher $EINOx$ of the Kroon (2022) [3] emissions inventory. An exact comparison cannot be made since it is not known which CO_2, CH_4 and fuel background scenario are used by Dahlmann, Grewe [19] and these can also still affect the result. The overall CO_2e ratio for CJF show good agreement (< 5% difference).

		This research	Dahlmann, 2023
**CJF**	CO_2e CO_2	1	1.0
	CO_2e H_2O	0.11	0.2

	CO_2e contrail	0.76	1.0
	CO_2e No_x	1.42	1.20
	CO_2e total	3.29	3.4
	$[gCO_2e/MJ]_{PtW}$	241.5	249.2
**SAF**	CO_2e CO_2	0	
	CO_2e H_2O	0.13	
	CO_2e contrail	0.55	
	CO_2e No_x	1.42	
	CO_2e total (incl. CO_2)	3.10	
	$[gCO_2e/MJ]_{PtW}$	147.75	
Reduction of $[gCO_2e/MJ]_{WtW}$ [%]		42.6%	
Reduction of $[gCO_2e/MJ]_{WtW}$		109.46	
non-CO_2 savings $[gCO_2e/MJ]_{PtW}$		20.46	

Supplementary Table 9: Comparison of ATR100 based CO_{2e} per specie & the total PtW CO_{2e} in g/MJ to Dahlmann et al. (2023) [19] for the Conventional Jet Fuel (CJF). Furthermore, the changes in WtW CO_{2e} caused by using 100% SAF are presented in both % and the absolute values.

SI 8 : Sensitivity Study

Various aspects and inputs of the model given in Supplementary Figure 1 are not in perfect agreement with literature and could wrongly affect the results. Therefore, we perform sensitivity study to investigate the effects of varying model setups and inputs on the CO_2e values, PtW results and WtW results for CJF and SAF. The elements that are varied are given in Supplementary Figure 5 along with the WtW results based on the ATR100 climate metric. In addition, Supplementary Figure 1 shows the CO_2 emissions savings (hashed yellow) and the non-CO_2 PtW emissions savings (hashed green) attained with 100% SAF. In Supplementary Table 8, the CO_{2e} values and GWP100 results of all perturbed simulations are also shown.

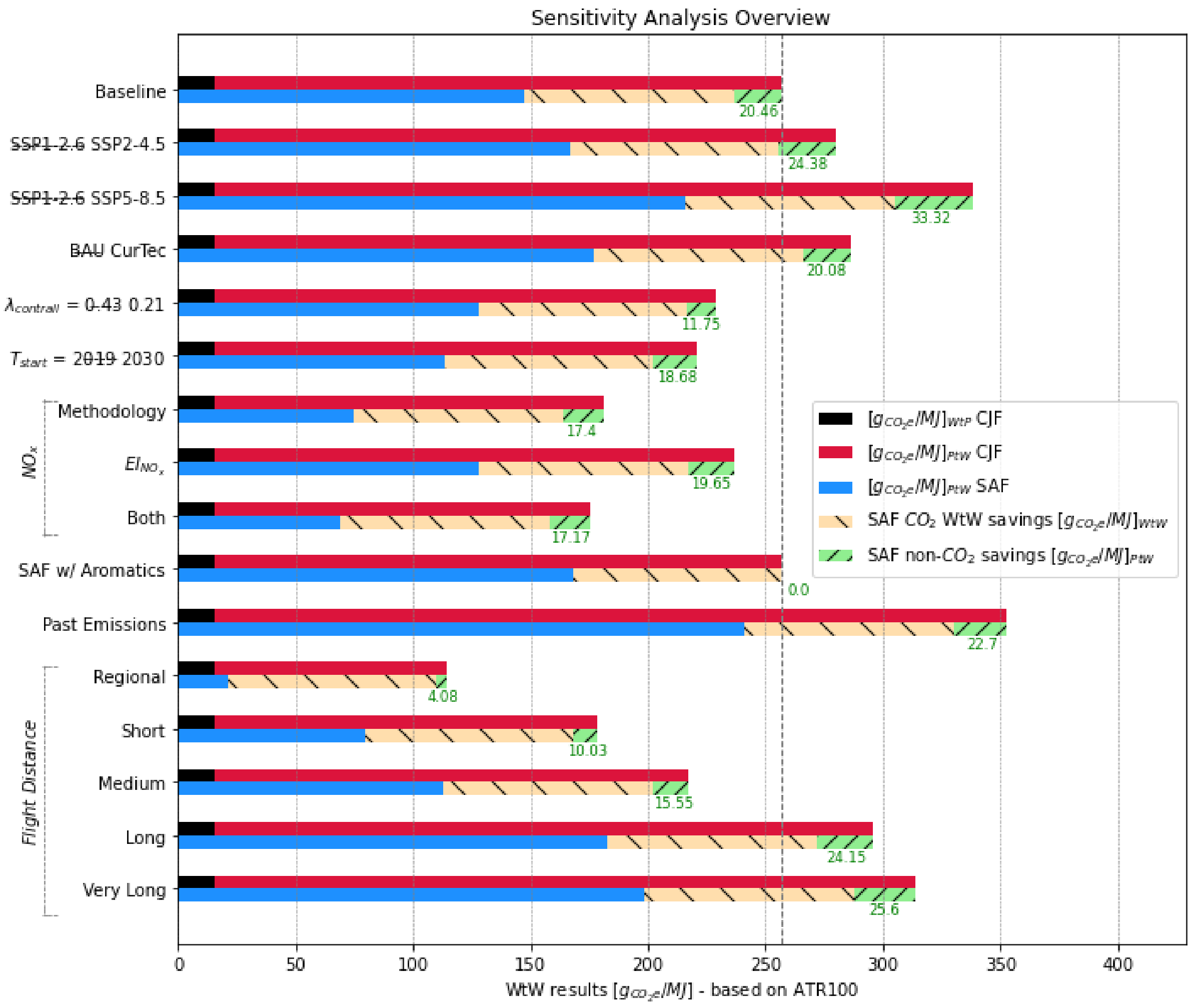

Supplementary Figure 5: Sensitivity Analysis Overview. The black and red bars denote the WtP and PtW emissions per MJ of CJF. The blue bars denote the PtW emissions (based on ATR100) per MJ of a perfect SAF type with 0 WtP emissions. The hashed yellow and green bars denote SAF CO_2 and non-CO_2 emissions reductions from an ideal (0 WtP) SAF respectively. The baseline simulation is shown on top and the effects of varying various model aspects on the results is shown.

As observed in Supplementary Figure 5, the climate impact reduction potential of SAF can be divided into two parts: (1) a lifecycle CO_2 emissions savings part which is already quantified in literature in previous lifecycle analyses and denoted by the light orange (left) hashed bars and a (2) non-CO_2 emissions reduction part denoted by the light green (right) dashed bars. (1) is independent of the model aspects and inputs since it solely depends on the SAF production pathway, whereas (2) does depend on the employed methodology. When changing model inputs or model aspects, the following happens:

1. If SSP2-4.5 or SSP5-8.5 are used CO_2 & CH_4 background scenario instead of SSP1-2.6, it is observed that CO_2e ratios and CJF & SAF PtW results become larger, since per unit of CO_2 will cause less warming than it previously would be due to the increase of the CO_2 concentrations in the atmosphere, meaning the CO_2 sink term is larger. Relative to CO_2, non-CO_2 effects will cause more warming, leading to an increase in the CO_2e factor. However, as non-CO_2 effects are more important and as SAF leads to non-CO_2 emissions reductions, the SAF non-CO_2 savings increase as well.
2. Changing the background fuel consumption scenario from BAU that assumes a steady increase in the aircraft efficiency over time, to CurTec that does not assume this increase in the aircraft efficiency over time (Grewe et al.,2021), leads to a higher CO_2e ratios and PtW results, since

in CurTec, more emissions (CO_2, NO_x, H_2O and traveled km) are made, but the additional temperature response of CO_2 is relatively small compared to other species due to saturation effects.

3. In a recent PhD thesis from Bickel [20], it was concluded that the climate sensitivity parameter of contrails $\lambda_{contrail}$ could be lower than initially thought. The main reason was that contrails are good at forcing radiation back to Earth, but not good at warming the Earth because of complicated cloud feedback. Hence, it studied what happens if this $\lambda_{contrail}$ in AirClim is set to 0.21 instead of 0.43- which is the AirClim default value from Ponater et al. (2006). The $CO_2e_{contrail}$ is decreased for both SAF as well as CJF, while other CO_2e ratios stay the same. Therefore, the PtW value is decreased. However, the emissions savings from SAF - that slightly mitigate contrails - are also reduced.
4. Instead of introducing the perturbation between scenario 1 (CJF) and 2 (SAF) from figure 1 in main in 2019 and integrating (using equation 1 in main) from 2019-2119, the perturbation can be introduced in 2030 and the integration can be done from 2030-2130. This results in the reduction of CO_2e and the PtW climate impact since the extra CO_2 emitted from 2019-2030 influences the radiative forcing and temperature responses, making the RF & temperature change caused by CO_2 from 2030-2130 higher.
5. The NO_x-O_3 estimated by the AirClim model is closer to the upper bound of the uncertainty range. To investigate the sensitivity of the model estimation, we change the methodology used to calculate NO_x radiative forcing in AirClim by using the methodology of [5], i.e., RF_{O3}(2018) = Tg_N(t) x 0.02514 [(mW/m^2)/(Tg$_N$/y)], making the O_3 RF proportional to nitrogen emissions. A similar change is made to the RF calculations of NO_x-related CH_4 and PMO in AirClim, resulting in lower CO_2e ratios and PtW results, but also slightly lower PtW non-CO_2 emission savings.
6. The NO_x emission index of the Kroon (2022) [3] emission inventory is higher than literature (e.g., Teoh et al., (2024) [4] and Lee et al., (2021)). By lowering this EI_{NOx} from 19 to 15.14, the CO_2e ratios and PtW results again decrease, but the PtW non-CO_2 savings also decrease.
7. When the emission inventory's NO_x emission index and the NO_x RF methodology are both lowered, the CO_2e ratios and PtW decrease even more substantially, but the non-CO_2 PtW emissions savings decrease only slightly since NO_x climate impact is reduced for both the CJF and SAF scenarios.
8. If a SAF type with aromatics is used, like Catalytic Hydrolysis Jet fuel (CHJ) or Fischer Tropsch with Aromatics (FT-SPK/A) [IATA], the reduction in soot and contrail climate impact will no longer occur and therefore, the exact same CO_2e ratio ought to be used for SAF as for CJF. This in turn leads to 0 PtW non-CO_2 emissions reductions.
9. When excluding past aviation emissions made from 1940-2018 by letting the simulation start in 2019 instead of in 1940, the CO_2 emitted from 1940-2018 is not allowed to build up and influence the radiative forcing and temperature responses of CO_2 from 2019-2130, leading to a weaker CO_2 RF and ΔT responses and higher CO_2e ratios. As a result, the PtW results are much higher, but the absolute reduction in emissions stays roughly the same as for the baseline simulation.
10. By dividing the Kroon (2022) [3] emissions inventory into 5 separate emissions inventories or clusters based on flight distance traveled (great circle distance or GCD), radiative forcing and temperature change responses of these individual clusters can be made and cluster-specific CO_2e ratios and PtW results can be derived, in a similar way as the flight distance dependent CO_2e ratios from Dahlmann (2023) [19] were derived. The following clusters are made: a regional cluster (GCD < 300nm), a short cluster (300< GCD <1000 nm), a medium cluster (1000< GCD <2000 nm), a long cluster (2000< GCD <4000 nm) and a very long cluster (GCD >4000 nm). As can be seen in figure SI 8, the PtW results (and therefore the CO_2e ratios) of the short cluster are much lower than those of the long & very long cluster, since flights in this cluster emit their emissions at lower altitudes where non-CO_2 emissions generally have a lower climate impact. However, the PtW non-CO_2 emissions savings per MJ of SAF are higher for the cluster with longer flights, meaning that it makes more sense to allocate SAF to longer flights with higher average flight altitudes and contrail climate impact.

		Baseline	SSP2-4.5	SSP5-8.5	CurTec	$\lambda_{contrail}$ = 0.21	T_{start} = 2030	NO_x			Aromatics	Past emissions excluded
								Method	EI $_{NOx}$	Both		
ATR100 CJF CO_2e ratio	CO_2e CO_2	1	1	1	1	1	1	1	1	1	1	1
	CO_2e H_2O	0.11	0.14	0.19	0.13	0.11	0.09	0.11	0.11	0.11	0.11	0.16
	CO_2e contrail	0.76	0.93	1.31	0.79	0.37	0.59	0.76	0.76	0.76	0.76	1.22
	CO_2e NO_x	1.42	1.53	1.90	1.77	1.42	1.12	0.38	1.14	0.30	1.42	2.22
	CO_2e total	3.29	3.60	4.40	3.69	2.90	2.80	2.26	3.02	2.18	3.29	4.60
$[gCO_{2e}/MJ]_{PtW}$ CJF		241.5	264.18	322.57	270.45	212.87	205.42	165.74	221.11	159.62	241.5	337.26
ATR100 SAF CO_2e ratio	CO_2e CO_2	0	0	0	0	0	0	0	0	0	0	0
	CO_2e H_2O	0.13	0.16	0.22	0.15	0.13	0.11	0.13	0.13	0.13	0.11	0.18
	CO_2e contrail	0.55	0.68	0.95	0.59	0.27	0.40	0.55	0.55	0.55	0.76	1.00
	CO_2e No_x	1.42	1.54	1.91	1.78	1.42	1.11	0.39	1.14	0.30	1.42	2.25
	CO_2e total (incl. CO_2)	3.10	3.37	4.08	3.52	2.82	2.62	2.07	2.83	1.99	3.29	4.44
$[gCO_{2e}/MJ]_{PtW}$ SAF		147.75	166.50	215.95	177.07	127.82	113.44	75.04	128.17	69.15	168.2	241.27
$[gCO_{2e}/MJ]_{WtW}$ [%-reduction]		42.6%	40.5%	36.2%	38.1%	44.1%	48.7%	58.6%	45.9%	60.6%	34.6%	31.6%
$[gCO_{2e}/MJ]_{WtW}$ [absolute-reduction]		109.46	113.38	122.32	109.08	100.75	107.68	106.40	108.65	106.17	89	111.70
PtW non-CO_2 savings [gCO_2e/MJ]		20.46	24.38	33.32	20.08	11.75	18.68	17.40	19.65	17.17	0	22.70

GWP100 CJF CO_2e ratio	CO_2e CO_2	1	1	1	1	1	1	1	1	1	1	1
	CO_2e H_2O	0.06	0.08	0.15	0.09	0.06	0.04	0.06	0.06	0.06	0.06	0.12
	CO_2e contrail	0.62	0.85	1.63	0.78	0.62	0.40	0.62	0.62	0.62	0.62	1.68
	CO_2e No_x	0.43	0.34	0.21	0.78	0.43	0.34	0.13	0.35	0.11	0.43	1.21
	CO_2e total	2.11	2.27	2.99	2.65	2.11	1.79	1.82	2.03	1.79	2.11	4.01
[gCO2e/MJ]_PtW CJF		154.6	166.7	219.1	194.0	154.6	131.1	133.1	148.7	131.1	154.6	293.6
GWP100 SAF CO_2e ratio	CO_2e CO_2	0	0	0	0	0	0	0	0	0	0	0
	CO_2e H_2O	0.07	0.09	0.18	0.10	0.07	0.05	0.07	0.07	0.07	0.06	0.13
	CO_2e contrail	0.52	0.71	1.37	0.65	0.52	0.33	0.52	0.52	0.52	0.62	1.38
	CO_2e No_x	0.44	0.35	0.22	0.79	0.44	0.35	0.13	0.35	0.11	0.43	1.22
	CO_2e total (incl. CO_2)	2.02	2.15	2.77	2.54	2.02	1.73	1.72	1.94	1.69	2.11	3.74
$[gCO_2e/MJ]_{PtW}$ SAF		71.5	80.8	123.9	107.9	71.5	51.4	50.4	65.7	48.5	81.3	192.3
$[gCO_2e/MJ]_{WtW}$ [%-reduction]		58.0%	55.7%	47.2%	48.6%	58.0%	65.0%	66.1%	60.0%	67.0%	52.2%	37.8%
$[gCO_2e/MJ]_{WtW}$ [absolute-reduction]		98.8	101.7	110.9	101.9	98.8	95.4	98.4	98.7	98.3	89	117.0
PtW non-CO_2 savings [gCO_2e/MJ]		9.8	12.7	21.9	12.9	9.8	6.4	9.4	9.7	9.3	0	28.0

Supplementary Table 10 sensitivity of the CO_2e changes with respect to different settings, for instance, the background scenarios, the climate efficiency, the starting year and the contents of aromatics in the fuel.

References

1. Grewe, V. and Stenke, A., *AirClim: an efficient tool for climate evaluation of aircraft technology.* Atmospheric Chemistry and Physics, 2008. 8(16): p. 4621-4639 DOI: 10.5194/acp-8-4621-2008.

2. Dahlmann, K., Grewe, V., Frömming, C., and Burkhardt, U., *Can we reliably assess climate mitigation options for air traffic scenarios despite large uncertainties in atmospheric processes?* Transportation Research Part D: Transport and Environment, 2016. 46: p. 40-55 DOI: https://doi.org/10.1016/j.trd.2016.03.006.

3. Kroon, R., *Aviation Emission Inventory: A contemporized bottom-up emission inventory for the year 2019*. 2022, Delft University of Technology.

4. Teoh, R., Engberg, Z., Shapiro, M., Dray, L., and Stettler, M.E.J., *The high-resolution Global Aviation emissions Inventory based on ADS-B (GAIA) for 2019–2021.* Atmos. Chem. Phys., 2024. 24(1): p. 725-744 DOI: 10.5194/acp-24-725-2024.

5. Lee, D.S., Fahey, D.W., Skowron, A., Allen, M.R., Burkhardt, U., Chen, Q., Doherty, S.J., Freeman, S., Forster, P.M., Fuglestvedt, J., Gettelman, A., De León, R.R., Lim, L.L., Lund, M.T., Millar, R.J., Owen, B., Penner, J.E., Pitari, G., Prather, M.J., Sausen, R., and Wilcox, L.J., *The contribution of global aviation to anthropogenic climate forcing for 2000 to 2018.* Atmospheric Environment, 2021: p. 117834 DOI: https://doi.org/10.1016/j.atmosenv.2020.117834.

6. Quadros, F.D.A., Snellen, M., Sun, J., and Dedoussi, I.C., *Global Civil Aviation Emissions Estimates for 2017–2020 Using ADS-B Data.* Journal of Aircraft, 2022. 59(6): p. 1394-1405 DOI: 10.2514/1.C036763.

7. Klenner, J., Muri, H., and Strømman, A.H., *Domestic and international aviation emission inventories for the UNFCCC parties.* Environmental Research Letters, 2024. 19(5): p. 054019 DOI: 10.1088/1748-9326/ad3a7d.

8. Moore, R.H., Thornhill, K.L., Weinzierl, B., Sauer, D., D'Ascoli, E., Kim, J., Lichtenstern, M., Scheibe, M., Beaton, B., Beyersdorf, A.J., Barrick, J., Bulzan, D., Corr, C.A., Crosbie, E., Jurkat, T., Martin, R., Riddick, D., Shook, M., Slover, G., Voigt, C., White, R., Winstead, E., Yasky, R., Ziemba, L.D., Brown, A., Schlager, H., and Anderson, B.E., *Biofuel blending reduces particle emissions from aircraft engines at cruise conditions.* Nature, 2017. 543(7645): p. 411-415 DOI: 10.1038/nature21420.

9. Kärcher, B., *Formation and radiative forcing of contrail cirrus.* Nature Communications, 2018. 9(1): p. 1824 DOI: 10.1038/s41467-018-04068-0.

10. Yu, F., Kärcher, B., and Anderson, B.E., *Revisiting Contrail Ice Formation: Impact of Primary Soot Particle Sizes and Contribution of Volatile Particles.* Environmental Science & Technology, 2024. 58(40): p. 17650-17660 DOI: 10.1021/acs.est.4c04340.

11. Teoh, R., Schumann, U., Voigt, C., Schripp, T., Shapiro, M., Engberg, Z., Molloy, J., Koudis, G., and Stettler, M.E.J., *Targeted Use of Sustainable Aviation Fuel to Maximize Climate Benefits.* Environmental Science & Technology, 2022. 56(23): p. 17246-17255 DOI: 10.1021/acs.est.2c05781.

12. Grewe, V., Gangoli Rao, A., Grönstedt, T., Xisto, C., Linke, F., Melkert, J., Middel, J., Ohlenforst, B., Blakey, S., Christie, S., Matthes, S., and Dahlmann, K., *Evaluating the climate*

impact of aviation emission scenarios towards the Paris agreement including COVID-19 effects. Nature Communications, 2021. 12(1): p. 3841 DOI: 10.1038/s41467-021-24091-y.

13. Burkhardt, U., Bock, L., and Bier, A., *Mitigating the contrail cirrus climate impact by reducing aircraft soot number emissions.* npj Climate and Atmospheric Science, 2018. 1(1): p. 37 DOI: 10.1038/s41612-018-0046-4.

14. Caiazzo, F., Agarwal, A., Speth, R.L., and Barrett, S.R.H., *Impact of biofuels on contrail warming.* Environmental Research Letters, 2017. 12(11): p. 114013 DOI: 10.1088/1748-9326/aa893b.

15. Bock, L. and Burkhardt, U., *Contrail cirrus radiative forcing for future air traffic.* Atmos. Chem. Phys., 2019. 19(12): p. 8163-8174 DOI: 10.5194/acp-19-8163-2019.

16. Märkl, R.S., Voigt, C., Sauer, D., Dischl, R.K., Kaufmann, S., Harlaß, T., Hahn, V., Roiger, A., Weiß-Rehm, C., Burkhardt, U., Schumann, U., Marsing, A., Scheibe, M., Dörnbrack, A., Renard, C., Gauthier, M., Swann, P., Madden, P., Luff, D., Sallinen, R., Schripp, T., and Le Clercq, P., *Powering aircraft with 100 % sustainable aviation fuel reduces ice crystals in contrails.* Atmos. Chem. Phys., 2024. 24(6): p. 3813-3837 DOI: 10.5194/acp-24-3813-2024.

17. Bier, A. and Burkhardt, U., *Impact of Parametrizing Microphysical Processes in the Jet and Vortex Phase on Contrail Cirrus Properties and Radiative Forcing.* Journal of Geophysical Research: Atmospheres, 2022. 127(23): p. e2022JD036677 DOI: https://doi.org/10.1029/2022JD036677.

18. Stratton, R.W., *Life cycle assessment of greenhouse gas emissions and non-CO_2 combustion effects from alternative jet fuels*. 2010, Massachusetts Institute of Technology: Massachusetts Institute of Technology. Department of Aeronautics and Astronautics.

19. Dahlmann, K., Grewe, V., Matthes, S., and Yamashita, H., *Climate assessment of single flights: Deduction of route specific equivalent CO2 emissions.* International Journal of Sustainable Transportation, 2023. 17(1): p. 29-40 DOI: https://doi.org/10.1080/15568318.2021.1979136.

20. Bickel, M., *Climate Impact of Contrail Cirrus*. 2023.
